# Exploring the views of infection consultants in England on a novel delinked funding model for antimicrobials: the SMASH study

**DOI:** 10.1093/jacamr/dlad091

**Published:** 2023-08-01

**Authors:** Ioannis Baltas, Mark Gilchrist, Eirini Koutoumanou, Malick M Gibani, James E Meiring, Akaninyene Otu, David Hettle, Ameeka Thompson, James R Price, Anna Crepet, Abolaji Atomode, Timothy Crocker-Buque, Dimitrios Spinos, Hudson Guyver, Matija Tausan, Donald Somasunderam, Maxwell Thoburn, Cathleen Chan, Helen Umpleby, Bethany Sharp, Callum Chivers, Devan Suresh Vaghela, Ronak J Shah, Jonathan Foster, Amy Hume, Christopher Smith, Ammara Asif, Dimitrios Mermerelis, Mohammad Abbas Reza, Dominic A Haigh, Thomas Lamb, Loucia Karatzia, Alexandra Bramley, Nikhil Kadam, Konstantinos Kavallieros, Veronica Garcia-Arias, Jane Democratis, Claire S Waddington, Luke S P Moore, Alexander M Aiken

**Affiliations:** Imperial College Healthcare NHS Trust, London, UK; Institute of Education, University College London, London, UK; Imperial College Healthcare NHS Trust, London, UK; Department of Infectious Disease, Faculty of Medicine, Imperial College London, London, UK; Population, Policy & Practice Research and Teaching Department, Great Ormond Street Institute of Child Health, University College London, London, UK; Department of Infectious Disease, Faculty of Medicine, Imperial College London, London, UK; Department of Infection, Immunity and Cardiovascular Disease, University of Sheffield, Sheffield, UK; Department of Microbiology, Leeds Teaching Hospitals NHS Trust, Leeds, UK; Department of Infection Sciences, Southmead Hospital, North Bristol NHS Trust, Bristol, UK; Department of Infection Sciences, Southmead Hospital, North Bristol NHS Trust, Bristol, UK; Brighton and Sussex Medical School, University of Sussex, Brighton, UK; University Hospitals Sussex NHS Foundation Trust, Brighton, UK; University Hospitals Sussex NHS Foundation Trust, Brighton, UK; Liverpool University NHS Foundation Trust, Liverpool, UK; Department of Microbiology, Royal Free London NHS Foundation Trust, London, UK; Department of ENT, Head and Neck Surgery, Gloucester Royal Hospital, Gloucestershire Hospitals NHS Foundation Trust, Gloucester, UK; James Paget University Hospitals NHS Foundation Trust, Norfolk, UK; Chelsea and Westminster Hospital NHS Foundation Trust, London, UK; Bart’s Health NHS Trust, London, UK; University Hospitals Birmingham NHS Foundation Trust, Birmingham, UK; University Hospitals Birmingham NHS Foundation Trust, Birmingham, UK; Hampshire Hospitals NHS Foundation Trust, Hampshire, UK; Nottingham University Hospitals NHS Trust, Nottingham, UK; Nottingham University Hospitals NHS Trust, Nottingham, UK; Norfolk and Norwich University Hospitals NHS Foundation Trust, Norwich, UK; Imperial College Healthcare NHS Trust, London, UK; Directorate of Pharmacy, The Newcastle upon Tyne Hospitals NHS Foundation Trust, Newcastle, UK; Directorate of Pharmacy, The Newcastle upon Tyne Hospitals NHS Foundation Trust, Newcastle, UK; Department of Infectious Disease, Faculty of Medicine, Imperial College London, London, UK; Hull University Teaching Hospitals NHS Trust, Hull, UK; Maidstone and Tunbridge Wells NHS Trust, Kent, UK; Northwest Anglia NHS Foundation Trust, Peterborough, UK; Manchester University NHS Foundation Trust, Manchester, UK; Nuffield Department of Clinical Medicine, University of Oxford, Oxford, UK; Lao-Oxford-Mahosot Hospital-Wellcome Trust Research Unit, Vientiane, Lao People’s Democratic Republic; Oxford University Hospitals NHS Trust, Oxford, UK; St George’s University Hospitals NHS Foundation Trust, London, UK; Mid and South Essex NHS Trust, Westcliff-on-Sea, UK; Faculty of Medicine, Imperial College London, London, UK; Hampshire Hospitals NHS Foundation Trust, Hampshire, UK; Wexham Park Hospital, Frimley Health NHS Foundation Trust, Slough, UK; Imperial College Healthcare NHS Trust, London, UK; Imperial College Healthcare NHS Trust, London, UK; Chelsea and Westminster Hospital NHS Foundation Trust, London, UK; Imperial College London, NIHR Health Protection Research Unit on Healthcare Associated Infections and Antimicrobial Resistance, London, UK; Department of Infectious Disease Epidemiology, London School of Hygiene and Tropical Medicine, London, UK

## Abstract

**Objectives:**

A novel ‘subscription-type’ funding model was launched in England in July 2022 for ceftazidime/avibactam and cefiderocol. We explored the views of infection consultants on important aspects of the delinked antimicrobial funding model.

**Methods:**

An online survey was sent to all infection consultants in NHS acute hospitals in England.

**Results:**

The response rate was 31.2% (235/753). Most consultants agreed the model is a welcome development (69.8%, 164/235), will improve treatment of drug-resistant infections (68.5%, 161/235) and will stimulate research and development of new antimicrobials (57.9%, 136/235). Consultants disagreed that the model would lead to reduced carbapenem use and reported increased use of cefiderocol post-implementation. The presence of an antimicrobial pharmacy team, requirement for preauthorization by infection specialists, antimicrobial stewardship ward rounds and education of infection specialists were considered the most effective antimicrobial stewardship interventions. Under the new model, 42.1% (99/235) of consultants would use these antimicrobials empirically, if risk factors for antimicrobial resistance were present (previous infection, colonization, treatment failure with carbapenems, ward outbreak, recent admission to a high-prevalence setting).

Significantly higher insurance and diversity values were given to model antimicrobials compared with established treatments for carbapenem-resistant infections, while meropenem recorded the highest enablement value. Use of both ‘subscription-type’ model drugs for a wide range of infection sites was reported. Respondents prioritized ceftazidime/avibactam for infections by bacteria producing OXA-48 and KPC and cefiderocol for those producing MBLs and infections with *Stenotrophomonas maltophilia*, *Acinetobacter* spp. and *Burkholderia cepacia*.

**Conclusions:**

The ‘subscription-type’ model was viewed favourably by infection consultants in England.

## Introduction

Antimicrobial resistance (AMR) remains a major threat to global health.^1,2^ Multiple interventions are required to control the AMR pandemic, including the development of novel antimicrobials to treat drug-resistant infections. Despite this threat, in 2020, only 41 new antimicrobials were in Phase 1 to 3 clinical trials, as they can be viewed by the pharmaceutical industry as commercially unattractive.^3,4^

To address the market failure for antimicrobials, various health policy interventions have been considered, including different forms of ‘push’ and ‘pull’ incentives.^5^ One of the proposed solutions is purchasing antimicrobials via a ‘subscription-type’ model. Under this funding model, a country pays a set annual payment (the ‘subscription’) to a company, for the right to secured access to a particular antimicrobial by a country’s healthcare system as required.^6^ In this way, the price paid for the antimicrobial is delinked from the volume sold. The main aim of this strategy is to stimulate research and development of new antimicrobials by guaranteeing a viable market for pharmaceutical companies.^7^

In July 2022, England became the first country to launch a fully delinked funding model for the purchase of antimicrobials in its publicly funded universal healthcare system, the NHS.^8^ The two antimicrobials selected to be included in the initial model pilot were ceftazidime/avibactam and cefiderocol. Each drug was prioritized for specific high-value clinical scenarios, which included OXA-48-producing infections for ceftazidime/avibactam and MBL-producing infections for cefiderocol.^9,10^ However, it was agreed that the use of these antimicrobials might be broader, including empirical use.^11^

The new funding model is set to run as a pilot for 3 years, with the option to extend the agreement to 10 years and include additional new antimicrobials. During the pilot period, there is an urgent need to collect evidence to evaluate the implementation and impact of the ‘subscription-type’ funding model on antimicrobial stewardship (AMS) and drug-resistant infections. This was clear during the procurement process, where multiple modelling assumptions had to be made based on limited data.^12^ There was particular uncertainty around the benefits of antimicrobials beyond the treatment of an individual patient, also known as the spectrum, transmission, enablement, diversity, insurance (STEDI) values of antimicrobials (Table 1).^13^ The subscription models for antibiotics in hospitals (SMASH) survey was designed to collect the views of infection consultants in England on important aspects of the implementation of the subscription-type payment model in the NHS.

**Table 1.** The STEDI values of antimicrobials^12,13^

Value	Benefits
Spectrum	The benefits of replacing other broad-spectrum antimicrobials that could be used to cure the same infection, with a narrower-spectrum antimicrobial.
Transmission	The benefits of avoiding the spread of the pathogen to the wider population if the patient with the infection responds promptly to treatment and is treated successfully.
Enablement	The benefits associated with enabling other treatments or procedures to take place, e.g. surgical and medical procedures that may not be possible if antimicrobials were not available to prevent or treat surgical site or post-procedure infections.
Diversity	The benefits of having a range of treatment options available to reduce selection pressure for resistance and to preserve the efficacy of existing antimicrobials.
Insurance	The value of having antimicrobials available in case of a sudden or major increase in the prevalence of infections with pathogens resistant to all other existing antimicrobials.

## Materials and methods

### Study population

The target population for the SMASH survey was all consultants with a UK General Medical Council Certificate of Completion of Training (CCT) in Infectious Diseases or Medical Microbiology working in an NHS acute hospital in England. Consultants with a CCT in Infectious Diseases or Medical Microbiology but working exclusively in Virology or Tropical Medicine were excluded.

### Survey conduction and administration

This was a cross-sectional survey in November and December 2022, 5–6 months after the launch of the ‘subscription-type’ funding model, across all NHS acute hospitals. Each participant was contacted up to three times and invited to complete the study questionnaire. Responses were not anonymous, as participants provided their professional e-mail addresses for their response to be recorded. Only fully completed, responses were recorded. A small financial incentive (10 GBP) in the form of a voucher was offered to all participants completing the study questionnaire.

### Questionnaire design

The questionnaire was developed by the study authors and the final version of the questionnaire is available in Appendix I (available as Supplementary data at *JAC-AMR* Online). The survey was administered using the online SurveyMonkey platform (Momentive Inc., USA).

### Statistical analysis

Analysis was performed in SPSS version 29 (IBM Corp, USA). Categorical variables were expressed as percentages with 95% CIs, continuous variables as means with 95% CIs or medians with IQRs, as appropriate. 95% CIs were calculated using 10 000 bootstrap samples. For Likert scales, agreement with the question was defined as the sum of participants who selected ‘agree’ or ‘strongly agree’ for their answers. Internal consistency of questions addressing similar themes was tested using Cronbach’s alpha.^14^ At the end of the survey, 10% of total responders were randomly selected to complete the survey a second time to examine consistency of responses using Cohen’s kappa.

### Ethics

Ethical approval for this study was provided by the Research Ethics Committee of the London School of Hygiene and Tropical Medicine (Ref. No. 28161/RR/29296). A more detailed description of the study methodology is provided in Appendix II.

## Results

### Participant characteristics

A total of 753 eligible consultants were identified in England during the study period, out of whom 235 completed the survey (31.2% response rate). At least one response was received from 66.2% of all NHS trusts (90/136). The average completion time was 32.5 min. A breakdown of participant characteristics is shown in Table 2 and a summary of all responses is given in Table S1. Responses were received from all seven UK Health Security Agency (UKHSA) regions but there were statistically significant different response rates across areas (Table 2). Overall, consultants with a CCT in Infectious Diseases and Medical Microbiology were more likely to answer the survey, while consultants with a CCT in Infectious Diseases and General Internal Medicine were less likely (Table 2). The median number of years from CCT for all consultants was 9 (IQR 4–16), while the average was 10.5 years (95% CI 9.4–11.5). Most consultants worked with both adult and paediatric patients (63.8%, 150/235), while 31.5% (74/235) only with adult and 4.7% (11/235) only with paediatric patients. Only 2.1% (5/235) of participants reported conflicts of interest when completing the study, all deemed significant. These responses were not excluded from the main analysis.

**Table 2.** Consultants surveyed

Category	Responders *n* (%)	Non-responders *n* (%)	Total *n* (%)	*P* value
All regions	235 (31.2)	518 (68.8)	753 (100)	0.03^a^
East of England	11 (17.2)	53 (82.8)	64 (100)	<0.05^b^
North West	27 (24.3)	84 (75.7)	111 (100)	>0.05^b^
London	41 (28.7)	102 (71.3)	143(100)	>0.05^b^
Midlands	39 (30.2)	80 (69.8)	119 (100)	>0.05^b^
South East	30 (35.7)	54 (64.3)	84 (100)	>0.05^b^
North East and Yorkshire	53 (37.1)	90 (62.9)	143 (100)	>0.05^b^
South West	34 (38.2)	55 (61.8)	89 (100)	>0.05^b^
Consultant regions	235 (31.2)	518 (68.8)	753 (100)	<0.001^a^
Infectious Diseases and General Internal Medicine	32 (22.9)	108 (77.1)	140 (100)	<0.05^b^
Medical Microbiology only	132 (29.3)	318 (70.7)	450 (100)	>0.05^b^
Infectious Diseases only	16 (29.6)	38 (70.4)	54 (100)	>0.05^b^
Infectious Diseases & Medical Microbiology	55 (50.5)	54 (49.5)	109 (100)	<0.05^b^

a Chi-squared across all categories.

b Denotes statistical significance among all pairwise comparisons. Pairwise comparisons are adjusted using the Bonferroni correction.

### General views on the subscription-type model

Only 58.3% (137/235) of consultants had heard of the ‘subscription-type’ funding model at the time of the study, while 63.8% (150/235) reported not receiving adequate information about it at the time of its launch. Most consultants agreed that the model was a welcome development (69.8%, 164/235) and would improve the ability of infection specialists to treat drug-resistant infections (68.5%, 161/235). Most felt that acquiring antimicrobials through the new model would increase their personal administrative workload compared with previous local methods for acquiring expensive antimicrobials (62.1%, 146/235), yet some consultants disagreed with this statement (15.7%, 37/235). The majority of consultants agreed that the new model would stimulate research and development of new antimicrobials (57.9%, 136/235), although a large proportion were unsure about it (34.9%, 82/235). Opinions about cost when selecting antimicrobials varied; it was an important factor for 46% of consultants (108/235), while others were unsure (24.3%, 57/235) or did not take it into account during their decision-making (29.8%, 70/235). Some consultants felt the model would improve cost-effectiveness in the management of drug-resistant infections (45.1%, 106/235), while some neither agreed nor disagreed (49.4%, 116/235).

### Application of the STEDI valuation principles to delinked and non-delinked antimicrobials

**Figure 1.**
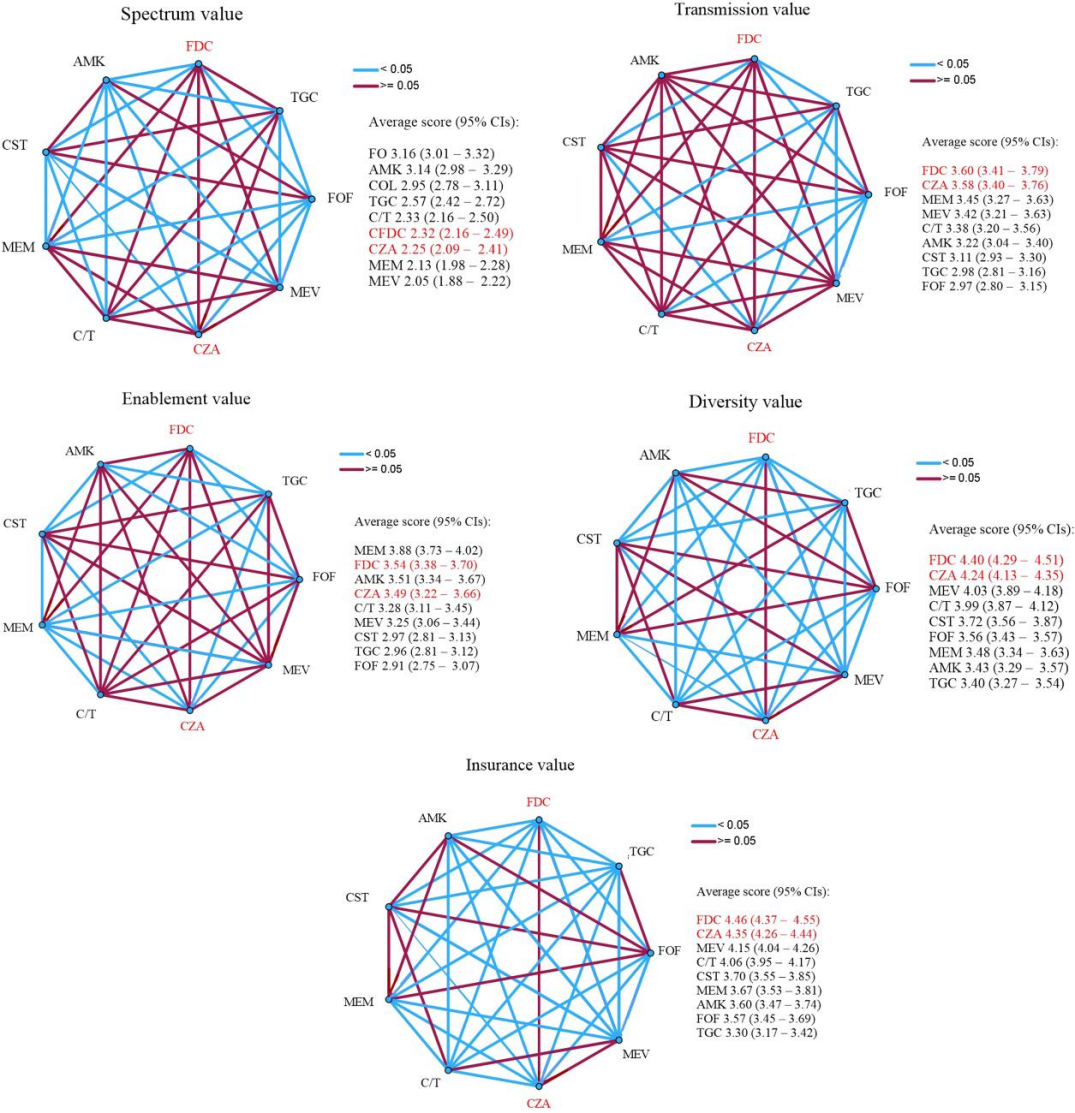
Pairwise comparisons (Kruskal–Wallis test), means and 95% CIs of values given by consultants to nine antimicrobials used for carbapenem-resistant infections to each of the STEDI values using a 5-point numerical scale (1—low value, 5—high value). Red lines connect antimicrobials without a statistically significant pairwise comparison (*P* ≥ 0.05), while blue lines connect antimicrobials with a statistically significant pairwise comparison (*P* < 0.05). All pairwise comparisons are adjusted using the Bonferroni correction. The 95% CIs were calculated using 10 000 bootstrap samples. CZA, ceftazidime/avibactam; FDC, cefiderocol; CST, colistin; TGC, tigecycline; FOF, fosfomycin; AMK, amikacin; MEM, meropenem; C/T, ceftolozane/tazobactam; MEV, meropenem/vaborbactam.

Consultants were asked to assess the STEDI values of the two antimicrobials introduced in the ‘subscription-type’ model using a 5-point numerical scale (1—low value, 5—high value) with seven other antimicrobials as comparators. The definitions of the STEDI values used are shown in Table 1. Overall, both ceftazidime/avibactam and cefiderocol were thought to have high insurance (mean 4.35/5 and 4.46/5, respectively) and diversity values (4.24/5 and 4.40/5) and their scores were statistically significantly higher than most comparators (Figure 1). High insurance and diversity values were also recorded for ceftolozane/tazobactam and meropenem/vaborbactam, which are other novel antimicrobials available for the treatment of carbapenem-resistant infections, although consultants were less likely to provide an answer for these drugs (Table S1). The two ‘subscription-type’ model antimicrobials also recorded moderate enablement value (3.49/5 and 3.54/5) and transmission value (3.58/5 and 3.60/5), with scores comparable to most other agents. The antimicrobials with the highest enablement value was meropenem (3.88/5), with a score statistically higher than all other agents apart from amikacin and cefiderocol (Figure 1). Mean transmission values were similar for most antimicrobials studied, except tigecycline and fosfomycin with lower recorded scores. A significantly higher proportion of consultants did not provide an assessment of transmission value for the selected antimicrobials (18.1%, 382/2115, Figure S1). Most antimicrobials included were thought to have low spectrum value. Statistically significant higher scores were recorded for fosfomycin, amikacin and colistin (Figure 1).

### Utilization of ‘subscription-type’ antimicrobials: frequency

There were significant differences in the confidence of consultants when using the antimicrobials introduced through the ‘subscription-type’ model compared with meropenem; 93.6% (220/235) reported being very or extremely confident in recognizing the clinical indications for treatment with meropenem, compared with 69.8% (164/235) for ceftazidime/avibactam and 54.5% (128/235) for cefiderocol (*P* < 0.001). This confidence correlated with levels of clinical usage; before the introduction of the ‘subscription-type’ model, most (87.2%, 205/235) consultants used meropenem on a daily or weekly basis, while use of the other two drugs was much less frequent (Table 3), with many consultants never having used cefiderocol (44.7%, 105/235) or ceftazidime/avibactam (10.6%, 25/235). In the first 5–6 months after the implementation of the model, there was significant perceived increase in the use of cefiderocol (*P* < 0.001), while use of meropenem (*P* = 0.30) and ceftazidime/avibactam (*P* = 0.14) remained unchanged (Table 3).

**Table 3.** Reported use of antimicrobials before and after the ‘subscription-type’ model

	Meropenem (*P* = 0.30)		Ceftazidime/avibactam (*P* = 0.14)		Cefiderocol (*P* < 0.001)	
	Before *n* (%)	After *n* (%)	Before *n* (%)	After *n* (%)	Before *n* (%)	After *n* (%)
Daily	106 (45.1)	97 (41.3)	0 (0)	0 (0)	0 (0)	1 (0.4)
Weekly	114 (48.5)	107 (45.5)	11 (4.7)	17 (7.2)	1 (0.4)	1 (0.4)
Monthly	14 (6)	24 (10.2)	69 (29.4)	68 (28.9)	25 (10.6)	34 (14.5)
Every 3–4 months	1 (0.4)	7 (3)	130 (55.3)	129 (54.9)	104 (44.3)	114 (48.5)
Never	0 (0)	0 (0)	25 (10.6)	21 (8.9)	105 (44.7)	85 (36.2)

*N* = 235. *P* value calculated from McNemar–Bowker test.

### Utilization of ‘subscription-type’ antimicrobials: patient pathways

**Figure 2.**
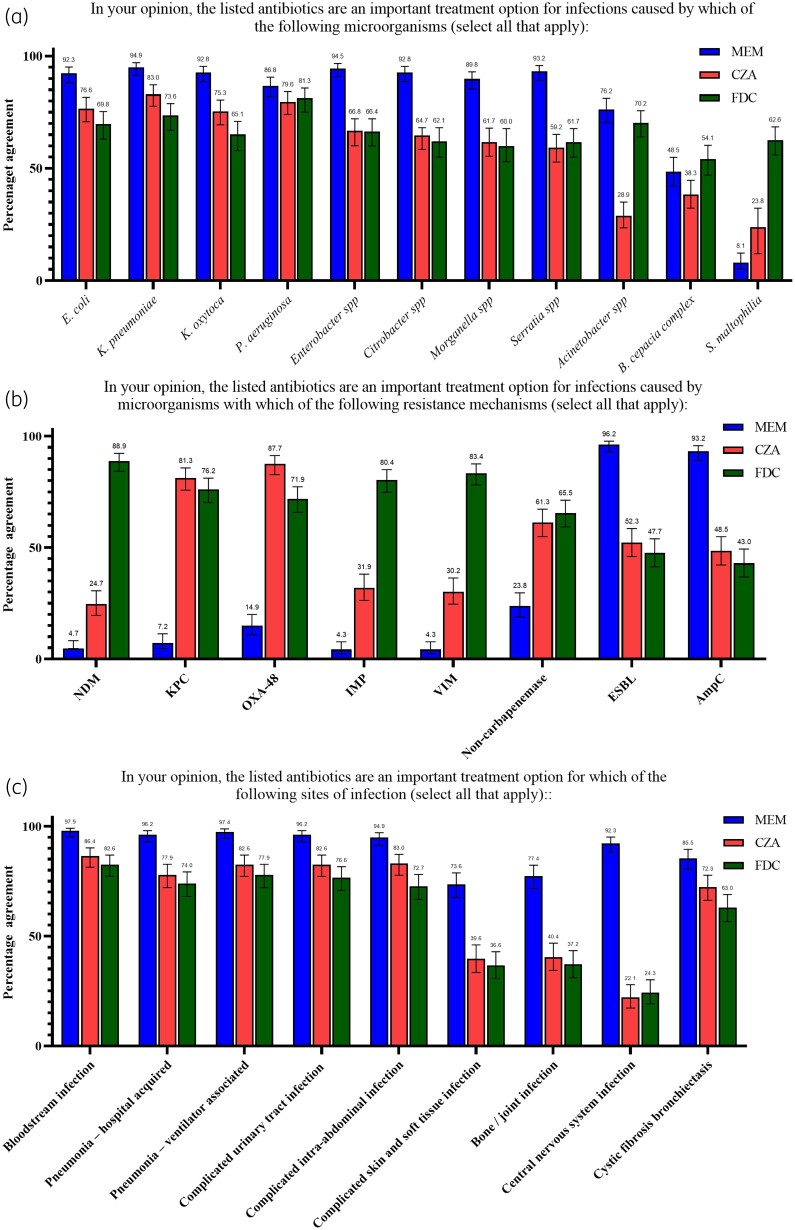
Proportions and 95% CIs of consultants who consider the listed antimicrobials an important treatment option for infections caused by the listed microorganism (a), infections caused by microorganisms with the listed resistance mechanism (b), and infections in the sites listed (c). 95% CIs were calculated using 10 000 bootstrap samples. MEM, meropenem; CZA, ceftazidime/avibactam; FDC, cefiderocol.

When asked about specific pathogens, consultants prioritized meropenem for the majority of Gram-negative infections, although many considered cefiderocol or ceftazidime/avibactam important secondary options, especially for *Pseudomonas aeruginosa* (Figure 2a). Cefiderocol was favoured for infections with *Acinetobacter* spp., *Burkholderia cepacia* and *Stenotrophomonas maltophilia*. Regarding resistance mechanisms, consultants prioritized treatment of all types of MBL producers with cefiderocol, although some would also consider treatment with ceftazidime/avibactam, which has demonstrated *in vitro* efficacy in combination with aztreonam (Figure 2b).^15^ Both ‘subscription-type’ antimicrobials were considered important treatment choices for infections by bacteria producing OXA-48 and KPC enzymes, with a preference for ceftazidime/avibactam. Treatment with meropenem was preferred for all infection sites, although many consultants would also consider treatment with ‘subscription-type’ model antimicrobials for many sites, including skin and soft tissue infection, bone and joint infections and cystic fibrosis/bronchiectasis (Figure 2c). Approximately half of the consultants considered ceftazidime/avibactam (48.5%, 114/235) and cefiderocol (46.4%, 109/235) to have a favourable toxicity profile compared with other treatments for carbapenem-resistant infections, while the rest were neutral. The majority would prioritize these drugs over colistin or aminoglycosides for the treatment of patients with renal impairment (80.9%, 190/235). Most consultants would consider off-licence treatment of paediatric patients (<18 years old) with ceftazidime/avibactam (86.4%, 203/235) or cefiderocol (76.6%, 180/235). This result was preserved in a sensitivity analysis excluding consultants working only with adults, and consultants not exclusively working with children (Table S1).

### Implication of the ‘subscription-type’ model for AMS

**Figure 3.**
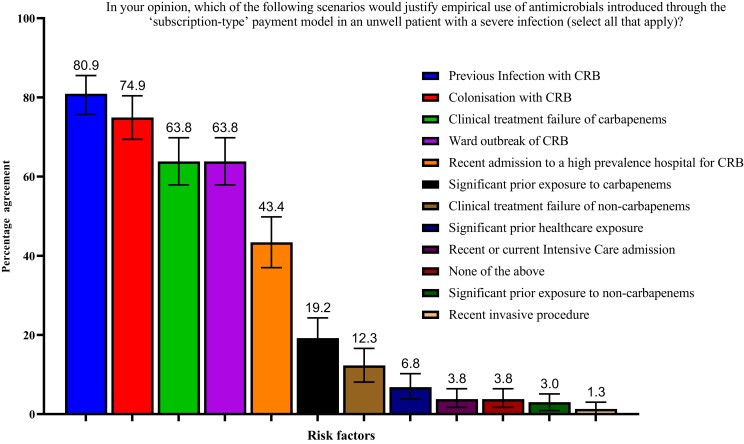
Proportions and 95% CIs of responders reporting that the listed risks factors would justify the empirical use of antimicrobials introduced through the ‘subscription-type’ model. 95% CIs were calculated using 10 000 bootstrap samples. CRB, carbapenem-resistant bacteria.

Regarding AMS, 45.5% (107/235) of consultants felt that the new funding model would not lead to reduced use of carbapenems, while a further 33.2% (78/235) were unsure. Measures to monitor usage and outcomes were seen positively, including the national registry (83.8%, 197/235) and Blueteq forms (70.2%, 165/235). Almost all consultants felt that infections by carbapenem-resistant bacteria would increase in the future (98.7%, 221/224). Consultants almost universally agreed (98.7%, 232/235) that preauthorization from an infection specialist should be required before the prescription of either ‘subscription-type’-model antimicrobials, either from a single consultant (39.2%, 92/235), a single consultant and a single AMS pharmacist (32.3%, 76/235) or from a multidisciplinary meeting (17.5%, 41/235). Ninety-nine of two hundred and thirty-five (42.1%) consultants would use the new antimicrobials empirically, particularly when risk factors for AMR were present (97.9%, 97/99). The most important risk factors justifying empirical use were felt to be previous infection with carbapenem-resistant bacteria (80.9%, 190/235), current colonization with carbapenem-resistant bacteria (74.9%, 176/235), clinical treatment failure of carbapenems (63.8%, 150/235), ward outbreak of carbapenem-resistant bacteria (63.8%, 150/235) and recent admission to a high-prevalence setting (43.4%, 102/235, Figure 3). When asked about the most effective AMS interventions using a 5-point numerical scale (1—not so effective, 5—very effective), consultants highly valued the presence of an antimicrobial pharmacy team (4.36/5), the requirement for preauthorization by infection specialists (4.22/5), AMS ward rounds (4.10/5) and education of infection specialists (4.07/5, Figure S1). Most consultants (73.2%, 172/235) agreed that *in vitro* susceptibility results predicted clinical outcomes in infections.

### Repeat survey

A total of 28 participants repeated the survey approximately 1 month after their original submission. Test/retest reliability results (Table S2) revealed moderate-to-substantial reliability. Cronbach’s alpha for questions addressing similar themes revealed good internal validity of results (Table S3).

## Discussion

We surveyed almost a third of all infection consultants in England on practical aspects of a novel delinked funding model for antimicrobials. Our main findings were that the model was viewed favourably and is likely to influence the utilization and stewardship of broad-spectrum novel antimicrobials in England.

To our knowledge, this is the first study that has directly assessed the STEDI values of antimicrobials by evaluating their end-user interpretation. Our results show that infection consultants in England consider novel antimicrobials to offer benefits beyond the treatment of individual patients, especially with regard to insurance and diversity value. Merit was also seen in terms of enablement value, which agrees with previous modelling in suggesting that rising AMR will soon threaten the safety and efficacy of surgical procedures and chemotherapy.^16^ Consultants felt that antimicrobials might have an effect on transmission. However, many did not provide an estimate for the transmission value, which suggests uncertainty. Indeed, most licensing studies focus their outcomes on clinical cure, therefore microbiological eradication and its subsequent effect on transmission is understudied. Interestingly, only insurance and enablement values were modelled during the evaluation process for the ‘subscription-type’ model.^12^

Our study also demonstrated that novel antimicrobials are likely to be used empirically, when reimbursed through a ‘subscription-type’ model. The top risk factors for empirical use reported by clinicians aligned with the ones currently suggested by the UKHSA.^9,10,12^ Greater use as empirical treatment may drive unnecessary resistance to these new therapies. This is compounded by challenges of accurately predicting which patients will develop carbapenem-resistant infections.^17,18^ Prospective monitoring and regulation of empirical treatment with ‘subscription-type’ antimicrobials via robust AMS programmes will be important to avoid this unintended consequence. Use of the two drugs for infection sites outside of their site-specific licensed indication is also to be expected, when limited treatment options are available.

Our data find that end-user opinion corroborates multiple modelling assumptions that were made during the procurement process for the model.^9,10^ Clinical trials for new antimicrobials typically adopt a non-inferiority design and enrol patients with infections susceptible to all agents used, both novel and comparator. This form of testing does not examine impacts in drug-resistant infections, where comparators drugs are liable to be less effective, thus precluding extrapolation of results, as treatment effects of the comparator are overestimated. Our study findings support that, in the absence of strong evidence, three-quarters of infection consultants in England think that using *in vitro* data to predict clinical outcomes in this scenario is reasonable, with the acceptance of some uncertainty. Consultants also agreed that rates of carbapenem-resistant infections are likely to increase in the future with a steady growth rate, which was an important modelling assumption made. Use of ceftazidime/avibactam and cefiderocol to avoid nephrotoxicity from colistin or aminoglycosides in patients with renal impairment is also likely, as well as the use of cefiderocol for *S. maltophilia* infections. Interestingly, many consultants would use cefiderocol for *Acinetobacter* spp., despite findings from the CREDIBLE study, which found suboptimal mortality rates for patients receiving this antimicrobial for this pathogen.^19^

Our study also highlights a potential caveat of the new ‘subscription-type’ funding model; consultants indicated that they would consider the two antimicrobials introduced through the ‘subscription-type’ model outside of high-value clinical scenarios, including the treatment of KPC infections and drug-resistant *P.aeruginosa*. We also showed that many consultants also consider cost in their decision-making for antimicrobials. These agents will be attractive treatment options for hospitals, as they will be significantly more affordable than other novel agents that have not been included in the delinked funding model. Yet, they may still not be the best treatment choices; treatment of KPC infections with ceftazidime/avibactam has been shown to lead to more emerging resistance than with meropenem/vaborbactam, while ceftolozane/tazobactam is a valuable treatment option for MDR *P.aeruginosa*, especially strains with high efflux pump activity.^20–22^ By limiting the agents included in the ‘subscription-type’ model to two, and providing them at functionally reduced cost compared with other novel antimicrobials, the ‘subscription-type’ model might have unintended consequences for AMR in England by reducing the diversity of agents.

The English ‘subscription-type’ model has been previously criticized for including two established antimicrobials, rather than truly novel agents.^23^ However, we found that most consultants in England rarely use these agents, while levels of resistance remain low.^24–28^ Therefore, it could be argued that ceftazidime/avibactam and cefiderocol are currently relatively novel to most clinicians and are suitable for acting as low utilization last-line treatment options in England, with relatively low levels of AMR compared with some countries.

Strengths of our study include achieving a high response rate of 31.2%, which is significantly higher than expected from an online survey of medical professionals, who are considered a difficult-to-reach group.^29^ For example, a similar national survey in the USA on outpatient parenteral therapy had a response rate of 8.5%.^30^ Participants provided their professional e-mail addresses, which were cross-referenced with local investigators, ensuring no contamination of survey results. Limitations include the lower response rates from specific regions and specialties, which affects sample representativeness. Many consultants had not heard of the model before, yet the questionnaire was sufficiently descriptive to allow respondents to give an informed opinion. At the time of the survey, ceftazidime/avibactam had been licensed for children up to the age of 3 months, therefore the wording of the question suggesting off-licence use in this group was inaccurate.

In conclusion, among end-user infection consultants in England, a ‘subscription-type’ model was viewed favourably and is likely to positively impact care. Delinked funding models are likely to affect the way physicians use antimicrobials, including indications and empirical use. Novel antimicrobials offer benefits beyond the treatment of individual patients, especially in terms of insurance and diversity value, and these metrics aid better understanding of value assessments for antimicrobials.

## Supplementary Material

dlad091_Supplementary_Data

## Transparency declarations

I.B. declares honoraria and investigator-initiated research funding from Shionogi B.V. during conduct of this project. M.G. is a member of the Pfizer Advisory Board. L.S.P.M. has consulted for or received speaker fees from bioMérieux, Pfizer, Eumedica, Kent Pharma, Umovis Lab, Shionogi, Pulmocide, Sumitovant and PhenUtest, and received research grants from the National Institute for Health and Care Research (NIHR), CW+ (i.e. the official charity of Chelsea and Westminster Hospital NHS Foundation Trust), InfectoPharm and LifeArc A.M.A. declares receipt of London School of Hygiene & Tropical Medicine salary contribution (4% full-time equivalent for 3 months) from Shionogi B.V. during conduct of this project. All other authors declare no conflicts of interest.

## Supplementary data

Appendices I and II, Figure S1 and Tables S1 to S3 are available as Supplementary data at *JAC-AMR* Online.
