# Supplementary material for: Exploring the views of infection consultants in England on a novel delinked funding model for antimicrobials: the SMASH study
Source: JAC Antimicrob Resist. 2023 Aug 1;5(4):dlad091. doi: 10.1093/jacamr/dlad091 (PMC10391702; doi:10.1093/jacamr/dlad091)
Supplement: dlad091_Supplementary_Data [file dlad091_supplementary_data.pdf]

Supplementary table 1: Summary of all study responses	
Question 1: Are you a Consultant in either Infectious Diseases, Medical Microbiology, Infectious Diseases and Medical Microbiology, or Infectious Diseases and General Internal Medicine currently working at the least 2 Programmed activities (PAs) per week or equivalent in a substantive or locum post of an NHS acute hospital?	
Yes	235/235 (100%)
No	0/235 (0%)
Question 2: Please select the specialty/combination of specialties that you currently hold a Certificate of completion of training (CCT) or Certificate of completion of Specialty training (CCST) from the General Medical Council (GMC), or equivalent in country of specialist training:	
Infectious Diseases	16/235 (6.8%)
Medical Microbiology	132/235 (56.2%)
Infectious Diseases and Medical Microbiology	55/235 (23.4%)
Infectious Diseases and General Internal Medicine	32/235 (13.6%)
Question 3: Please state the year that you received the above CCT/CCST (or overseas equivalent):	
Mean: 10.5 (95% CI: 9.4 - 11.5)	Median: 9 (IQR 4 – 16)
Question 4: Please indicate the NHS region you are currently working in:	
East of England	11/235 (4.7%)
London	41/235 (17.5%)
Midlands	39/235 (16.6%)
North East and Yorkshire	53/235 (22.6%)
North West	27/235 (11.5%)
South East	30/235 (12.8%)
South West	34/235 (14.5%)
Question 5: Please provide the name of the hospital/hospitals you currently work in (this will only be used to assess survey sample representativeness and not to identify you, please avoid abbreviations):	
Responses to this question were not published.	
Question 6: Please indicate the type of patients you currently work with:	
Adult	74/235 (31.5%)
Paediatric	11/235 (4.7%)
Both	150/235 (63.8%)
Question 7: Do you have any relevant conflicts of interest when completing this survey (this does not preclude you from completing the survey)?	
Yes	5/235 (2.1%)
No	230/235 (97.9%)
Question 8: Please declare your conflicts of interest	
Responses to this question were not published.	
Question 9: Before completing this survey, had you previously heard about the 'subscription-type' payment model for antimicrobial drugs?	
Yes	137/235 (58.3%, 51.9% – 64.7%)

No	98/235 (41.7%, 35.3% – 48.1%)
Question 10: The ‘subscription-type’ payment model is a welcome development in the management of drug-resistant infections.	
Strongly Agree	37/235 (15.7%, 11.5% – 20.4%)
Agree	127/235 (54%, 47.7% – 60.4%)
Neither Agree or Disagree	66/235 (28.2%, 22.6 – 33.6)
Disagree	5/235 (2.1%, 0.4% - 4.3%)
Strongly Disagree	0/235 (0%)
Question 11: Access to novel antimicrobials through the ‘subscription-type’ payment model will improve the ability of infection specialists to treat drug-resistant infections.	
Strongly Agree	30/235 (12.8%, 8.5% – 17%)
Agree	131/235 (55.7%, 49.4% – 62.1%)
Neither Agree or Disagree	58/235 (24.7%, 19.1% – 30.2%)
Disagree	15/235 (6.4%, 3.4% – 9.8%)
Strongly Disagree	1/235 (0.4%, 0% – 1.3%)
Question 12: Cost is a significant consideration for me when using/recommending the use of specific antimicrobials:	
Strongly Agree	17/235 (7.2%, 4.3% – 10.6%)
Agree	91/235 (38.7%, 32.8% – 45.1%)
Neither Agree or Disagree	57/235 (24.3%, 18.7% – 29.8%)
Disagree	59/235 (25.1%, 19.6% – 30.6%)
Strongly Disagree	11/235 (4.7%, 2.1% – 7.7%)
Question 13: Access to novel antimicrobials through the ‘subscription-type’ payment model will improve cost-effectiveness in the management of drug-resistant infections.	
Strongly Agree	19/235 (8.1%, 4.7% – 11.5%)
Agree	87/235 (37%, 31.1% – 43%)
Neither Agree or Disagree	116/235 (49.4%, 43% – 55.7%)
Disagree	11/235 (4.7%, 2.1% – 7.7%)
Strongly Disagree	2/235 (0.8%, 0% - 2.1%)
Question 14: Prescribing indications for antimicrobials supplied through the ‘subscription-type’ payment model are being monitored through the use of Blueteq forms, while outcomes of patients are being monitored through the UK Antimicrobial Registry. Both monitoring modalities are currently voluntary.<br>This will increase your personal administrative workload in the management of drug-resistant infections compared to previous local arrangements (e.g. completion of non-formulary or unlicensed drug requests, requests for financial approval for excess drug acquisition costs) for acquiring these antimicrobials.	
Strongly Agree	35/235 (14.9%, 10.6% – 19.6%)
Agree	111/235 (47.2%, 40.9% – 53.6%)
Neither Agree or Disagree	52/235 (22.1%, 17% – 27.7%)
Disagree	33/235 (14.1%, 9.8% – 18.7%)
Strongly Disagree	4/235 (1.7%, 0.4% - 3.4%)
Question 15: The ‘subscription-type’ payment model will stimulate research and development of new antimicrobials by pharmaceutical companies.	

Strongly Agree	29/235 (12.3%, 8.1% – 16.6%)
Agree	107/235 (45.5%, 39.1% – 51.9%)
Neither Agree or Disagree	82/235 (34.9%, 28.9% – 41.3%)
Disagree	15/235 (6.4%, 3.4% – 9.8%)
Strongly Disagree	2/235 (0.9%, 0% – 2.1%)
Question 16: The term "insurance value" refers to the value of having antibiotics available in case of a sudden or major increase in the prevalence of infections with pathogens resistant to all other existing antibiotics. On a scale of 1 – 5, please assess the insurance value of the following antibiotics:	
Ceftazidime-avibactam (N = 230)	4.35 (4.26 – 4.44)
Cefiderocol (N = 223)	4.46 (4.37 – 4.55)
Colistin (N = 231)	3.70 (3.55 – 3.85)
Tigecycline (N = 231)	3.30 (3.17 – 3.42)
Fosfomycin (N = 231)	3.57 (3.45 – 3.69)
Amikacin (N = 230)	3.60 (3.47 – 3.74)
Meropenem (N =232)	3.67 (3.53 – 3.81)
Ceftolozane-tazobactam (N =221)	4.06 (3.95 – 4.17)
Meropenem-vaborbactam (N = 208)	4.15 (4.04 – 4.26)
Question 17: The term "enablement value" refers to the benefits associated with enabling other treatments or procedures to take place, e.g., surgical and medical procedures that may not be possible if antimicrobials were not available to prevent or treat surgical site or post-procedure infections. On a scale of 1 – 5, please assess the enablement value of the following antibiotics, given the current prevalence of resistant infections:	
Ceftazidime-avibactam (N = 220)	3.49 (3.22 – 3.66)
Cefiderocol (N = 212)	3.54 (3.38 – 3.70)
Colistin (N = 221)	2.97 (2.81 – 3.13)
Tigecycline (N = 221)	2.96 (2.81 – 3.12)
Fosfomycin (N = 218)	2.91 (2.75 – 3.07)
Amikacin (N = 221)	3.51 (3.34 – 3.67)
Meropenem (N = 222)	3.88 (3.73 – 4.02)
Ceftolozane-tazobactam (N = 210)	3.28 (3.11 – 3.45)
Meropenem-vaborbactam (N = 196)	3.25 (3.06 – 3.44)
Question 18: The term "transmission value" refers to the benefits of avoiding the spread of the pathogen to the wider population if the patient with the infection responds promptly to treatment and is treated successfully. On a scale of 1 – 5, please assess the transmission value of the following antibiotics:	
Ceftazidime-avibactam (N = 194)	3.58 (3.40 – 3.76)
Cefiderocol (N = 190)	3.60 (3.41 – 3.79)
Colistin (N = 197)	3.11 (2.93 – 3.30)
Tigecycline (N = 196)	2.98 (2.81 – 3.16)
Fosfomycin (N = 196)	2.97 (2.80 – 3.15)
Amikacin (N = 197)	3.22 (3.04 – 3.40)
Meropenem (N = 198)	3.45 (3.27 – 3.63)
Ceftolozane-tazobactam (N = 189)	3.38 (3.20 – 3.56)

Meropenem-vaborbactam (N = 176)		3.42 (3.21 – 3.63)
Question 19: The term “diversity value” refers to the benefits of having a range of treatment options available to reduce selection pressure for resistance and to preserve the efficacy of existing antimicrobials. For example, a new antibiotic, typically effective against Vancomycin-resistant Staphylococcus aureus, where alternative treatment options are limited, would have high diversity value, while a new antibiotic typically effective against Group A streptococcus, where multiple alternative treatment options are available, would have low diversity value. On a scale of 1 – 5, please assess the diversity value of having/adding the following antibiotics to your existing hospital formulary:		
Ceftazidime-avibactam (N = 226)		4.24 (4.13 – 4.35)
Cefiderocol (N = 219)		4.40 (4.29 – 4.51)
Colistin (N = 224)		3.72 (3.56 – 3.87)
Tigecycline (N = 224)		3.40 (3.27 – 3.54)
Fosfomycin (N = 226)		3.56 (3.43 – 3.57)
Amikacin (N = 225)		3.43 (3.29 – 3.57)
Meropenem (N = 225)		3.48 (3.34 – 3.63)
Ceftolozane-tazobactam (N = 216)		3.99 (3.87 – 4.12)
Meropenem-vaborbactam (N = 191)		4.03 (3.89 – 4.18)
Question 20: The term “spectrum value” refers to the benefits of replacing other broad-spectrum antibiotics that could be used to cure the same infection, with a narrower spectrum antibiotic. For example, an antibiotic with a spectrum similar to flucloxacillin would be considered to have high spectrum value, while an antibiotic with a spectrum similar to piperacillin/tazobactam would be considered to have low spectrum value. On a scale of 1 – 5, please assess the spectrum value of the following antibiotics for replacing broad-spectrum agents:		
Ceftazidime-avibactam (N = 225)		2.25 (2.09 – 2.41)
Cefiderocol (N = 220)		2.32 (2.16 – 2.49)
Colistin (N = 225)		2.95 (2.78 – 3.11)
Tigecycline (N = 223)		2.57 (2.42 – 2.72)
Fosfomycin (N = 225)		3.16 (3.01 – 3.32)
Amikacin (N = 224)		3.14 (2.98 – 3.29)
Meropenem (N = 226)		2.13 (1.98 – 2.28)
Ceftolozane-tazobactam (N = 220)		2.33 (2.16 –2.50)
Meropenem-vaborbactam (N = 210)		2.05 (1.88 – 2.22)
Questions 21: Please indicate how confident you feel with recognizing the type of infections that would require treatment with the following antibiotics:		
Meropenem	Not at all confident	0/235 (0%)
	Not so confident	1/235 (0.4%, 0% – 1.3%)
	Somewhat confident	14/235 (6%, 3% – 8.9%)
	Very confident	114/235 (48.5%, 38.7% – 51.5%)
	Extremely confident	106/235 (45.1%, 38.7% – 51.5%)
Ceftazidime-avibactam	Not at all confident	1/235 (0.4%, 0% – 1.3%)
	Not so confident	14/235 (6%, 3% – 8.9%)
	Somewhat confident	56/235 (23.8%, 18.7% – 29.4%)
	Very confident	121/235 (51.5%, 45.1% – 57.9%)

	Extremely confident	43/235 (18.3%, 13.6% – 23.4%)
Cefiderocol	Not at all confident	5/235 (2.1%, 0.4% – 4.3%)
	Not so confident	28/235 (11.9%, 8.1% – 16.2%)
	Somewhat confident	74/235 (31.5%, 26% – 37.4%)
	Very confident	96/235 (40.9%, 34.5% – 46.8%)
	Extremely confident	32/235 (13.6%, 9.4% – 18.3%)
Question 22: In your routine clinical practice before the introduction of the ‘subscription-type’ payment model (ie before 1st July 2022), which of the following options best represents the frequency that you used/ recommended use of the following antibiotics?		
Meropenem	Never	0/235 (0%)
	Every 3 – 4 months	4/235 (1.7%, 0.4% – 3.4%)
	Monthly	26/235 (11.1%, 7.2% – 15.3%)
	Weekly	107/235 (45.5%, 39.1% – 51.9%)
	Daily	98 (41.7%, 35.3% – 48.1%)
Ceftazidime-avibactam	Never	25/235 (10.6%, 6.8% – 14.9%)
	Every 3 – 4 months	130/235 (55.3%, 48.9% – 61.7%)
	Monthly	69/235 (29.4%, 23.4% – 35.3%)
	Weekly	11/235 (4.7%, 2.1% – 7.7%)
	Daily	0/235 (0%)
Cefiderocol	Never	105/235 (44.7%, 38.3 – 51.1%)
	Every 3 – 4 months	104/235 (44.3%, 37.9% – 50.6%)
	Monthly	25/235 (10.6%, 6.8% – 14.9%)
	Weekly	1/235 (0.4%, 0% – 1.3%)
	Daily	0/235 (0%)
Question 23: In your routine current clinical practice after the introduction of the ‘subscription-type’ payment model (ie after 1st July 2022), which of the following options best represents the frequency that you use/ recommend use of the following antibiotics?		
Meropenem	Never	0/235 (0%)
	Every 3 – 4 months	7/235 (3%, 0.9% - 5.1%)
	Monthly	24/235 (10.2%, 6.4% – 14%)
	Weekly	107/235 (45.5%, 39.1% – 51.9%)
	Daily	97/235 (41.3%, 34.9% – 47.7%)
Ceftazidime-avibactam	Never	21/235 (8.9%, 5.5% – 12.8%)
	Every 3 – 4 months	129/235 (54.9%, 48.5% – 61.3%)
	Monthly	68/235 (28.9%, 23.4% – 34.9%)
	Weekly	17/235 (7.2%, 4.3% – 10.6%)
	Daily	0/235 (0%)
Cefiderocol	Never	85/235 (36.2%, 30.2% – 42.6%)
	Every 3 – 4 months	114/235 (48.5%, 42.1% – 54.9%)
	Monthly	34/235 (14.5%, 10.2% – 19.1%)
	Weekly	1/235 (0.4%, 0% – 1.3%)
	Daily	1/235 (0.4%, 0% – 1.3%)

Question 24: In your opinion, the listed antibiotics are an important treatment option for infections caused by which of the following microorganisms (select all that apply):		
Meropenem	*Escherichia coli*	217/235 (92.3%, 88.2% – 95.1%)
	Klebsiella pneumoniae	223/235 (94.9%, 91.3% – 97.1%)
	Klebsiella oxytoca	218/235 (92.8%, 88.7% – 95.4%)
	Pseudomonas aeruginosa	204/235 (86.8%, 81.9% – 90.6%)
	Enterobacter spp.	222/235 (94.5%, 90.8% – 96.7%)
	Citrobacter spp.	218/235 (92.8%, 88.7% – 95.4%)
	Morganella spp.	211/235 (89.8%, 85.3% – 93%)
	Serratia spp.	219/235 (93.2%, 89.2% – 95.8%)
	Acinetobacter spp.	179/235 (76.2%, 70.3% – 81.2%)
	Burkholderia cepacia complex	114/235 (48.5%, 42.2% – 54.9%)
	Stenotrophomonas maltophilia	19/235 (8.1%, 5.2% –12.3%)
Ceftazidime-avibactam	*Escherichia coli*	180/235 (76.6%, 70.8% – 81.6%)
	Klebsiella pneumonia	195/235 (83%, 77.7% – 87.2%)
	Klebsiella oxytoca	177/235 (75.3%, 69.4% – 80.4%)
	Pseudomonas aeruginosa	187/235 (79.6%, 74% – 84.2%)
	Enterobacter spp.	157/235 (66.8%, 60.1% – 72.1%)
	Citrobacter spp.	152/235 (64.7%, 58.4% – 68.1%)
	Morganella spp.	145/235 (61.7%, 55.4% – 67.9%)
	Serratia spp.	139/235 (59.2%, 52.8% – 65.2%)
	Acinetobacter spp.	68/235 (28.9%, 23.5% – 35%)
	Burkholderia cepacia complex	90/235 (38.3%, 32.3% – 44.7%)
	Stenotrophomonas maltophilia	56/235 (23.8%, 18.8% – 32.3%)
Cefiderocol	*Escherichia coli*	164/235 (69.8%, 63.4% – 75.3%)
	Klebsiella pneumonia	173/235 (73.6%, 67.6% – 78.8%)
	Klebsiella oxytoca	153/235 (65.1%, 58.8% – 70.9%)
	Pseudomonas aeruginosa	191/235 (81.3%, 75.8% – 85.8%)
	Enterobacter spp.	156/235 (66.4%, 60.1% – 72.1%)
	Citrobacter spp.	146/235 (62.1%, 55.8% – 68.1%)
	Morganella spp.	141/235 (60%, 53.6% – 67.7%)
	Serratia spp.	145/235 (61.7%, 55.4% – 67.7%)
	Acinetobacter spp.	165/235 (70.2%, 64.1% – 75.7%)

	Burkholderia cepacia complex	127/235 (54.1%, 47.7% – 60.3%)
	Stenotrophomonas maltophilia	147/235 (62.6%, 56.2% – 68.5%)
Question 25: In your opinion, the listed antibiotics are an important treatment option for infections caused by microorganisms with which of the following resistance mechanisms (select all that apply):		
Meropenem	NDM	11/235 (4.7%, 2.6% – 8.2%)
	KPC	17/235 (7.2%, 4.6% – 11.3%)
	OXA-48	35/235 (14.9%, 10.9% – 20%)
	IMP	10/235 (4.3%, 2.3% – 7.7%)
	VIM	10/235 (4.3%, 2.3% – 7.7%)
	Non-carbapenemase carbapenem resistance	56/235 (23.8%, 18.8% – 29.7%)
	ESBL	226/235 (96.2%, 92.9% – 97.8%)
	AmpC	219/235 (93.2%, 89.2% – 95.8%)
Ceftazidime-avibactam	NDM	58/235 (24.7%, 19.6% – 30.6%)
	KPC	191/235 (81.3%, 75.8% - 85.8%)
	OXA-48	206/235 (87.7%, 82.8% – 91.3%)
	IMP	75/235 (31.9%, 26.3% – 38.1%)
	VIM	71/235 (30.2%, 24.7% – 36.4%)
	Non-carbapenemase carbapenem resistance	144/235 (61.3%, 54.9% – 67.3%)
	ESBL	123/235 (52.3%, 46% – 58.6%)
	AmpC	114/235 (48.5%, 42.2 – 54.9%)
Cefiderocol	NDM	209/235 (88.9%, 84.3% – 92.3%)
	KPC	179/235 (76.2%, 70.3% - 81.2%)
	OXA-48	169/235 (71.9%, 65.8% – 77.3%)
	IMP	189/235 (80.4%, 74.9% – 85%)
	VIM	196/235 (83.4%, 78.1 – 87.6%)
	Non-carbapenemase carbapenem resistance	154/235 (65.5%, 59.3% – 71.3%)
	ESBL	112/235 (47.7%, 41.4% – 54%)
	AmpC	101/235 (43%, 36.8% – 49.4%)
Key - NDM: New Delhi Metallo-beta-lactamase, KPC: Klebsiella pneumoniae carbapenemase, OXA-48: Oxacillinase-48, IMP: Imipenemase, VIM: Verona integrated-encoded Metallo-beta-lactamase, ESBL: Extended-spectrum β-lactamase, AmpC: Ampicillinase C.		
Question 26: In your opinion, the listed antibiotics are an important treatment option for which of the following sites of infection (select all that apply):		
Meropenem	Bloodstream infection	230/235 (97.9%, 95.1% – 99.1%)

	Pneumonia – hospital acquired	226/235 (96.2%, 92.9% – 98%)
	Pneumonia – ventilator associated	229/235 (97.4%, 94.5% – 98.8%)
	Complicated urinary tract infection	226/235 (96.2%, 92.9% – 98%)
	Complicated intra-abdominal infection	223/235 (94.9%, 91.3% – 97.1%)
	Complicated skin and soft tissue infection	173/235 (73.6%, 67.6% – 78.8%)
	Bone / joint infection	182/235 (77.4%, 71.7% – 82.3%)
	Central nervous system infection	217/235 (92.3%, 88.2% –95.1%)
	Cystic fibrosis bronchiectasis	201/235 (85.5%, 80.5% – 89.5%)
Ceftazidime-avibactam	Bloodstream infection	203/235 (86.4%, 81.4% – 90.2%)
	Pneumonia – hospital acquired	183/235 (77.9%, 72.1% – 82.7%)
	Pneumonia – ventilator associated	194/235 (82.6%, 77.2% – 86.9%)
	Complicated urinary tract infection	194/235 (82.6%, 77.2% – 86.9%)
	Complicated intra-abdominal infection	195/235 (83%, 77.7% – 87.2%)
	Complicated skin and soft tissue infection	93/235 (39.6%, 33.5% – 46%)
	Bone / joint infection	95/235 (40.4%, 34.4% – 46.8%)
	Central nervous system infection	52/235 (22.1%, 17.3% – 27.9%)
	Cystic fibrosis bronchiectasis	170/235 (72.3%, 66.3% –77.7%)
Cefiderocol	Bloodstream infection	194/235 (82.6%, 77.2% – 86.9%)
	Pneumonia – hospital acquired	174/235 (74%, 68.1% – 79.2%)
	Pneumonia – ventilator associated	183/235 (77.9%, 72.1% – 82.7%)
	Complicated urinary tract infection	180/235 (76.6%, 70.8% – 81.6%)
	Complicated intra-abdominal infection	171/235 (72.7%, 66.7% – 78.1%)

	Complicated skin and soft tissue infection	86/235 (36.6%, 30.7% – 42.9%)
	Bone / joint infection	87/235 (37.2%, 31.1% – 43.4%)
	Central nervous system infection	57/235 (24.3%, 19.2% – 30.1%)
	Cystic fibrosis bronchiectasis	148/235 (63%, 56.6% – 68.9%)
Question 27: Ceftazidime/avibactam is associated with less treatment-related toxicity than other locally available antibiotic regimens for the treatment of OXA-48-producing carbapenem-resistant infections.		
Strongly Agree		17/235 (7.2%, 4.3% – 10.6%)
Agree		97/235 (41.3%, 34.9% – 47.7%)
Neither Agree or Disagree		112/235 (47.7%, 41.3% – 54%)
Disagree		8/235 (3.4%, 1.3% – 6%)
Strongly Disagree		1/235 (0.4%, 0% – 1.3%)
Question 28: Cefiderocol is associated with less treatment-related toxicity than other locally available antibiotic regimens for the treatment of metallo-beta-lactamase-producing carbapenem-resistant infections.		
Strongly Agree		22/235 (9.4%, 6% – 13.2%)
Agree		87/235 (37%, 31.1% – 43%)
Neither Agree or Disagree		118/235 (50.2%, 43.8% – 56.6%)
Disagree		7/235 (3%, 0.9% – 5.5%)
Strongly Disagree		1/235 (0.4%, 0% – 1.3%)
Question 29: Off-licence treatment of paediatric patients (<18 years old) with severe infections due to Gram-negative bacteria with limited treatment options, with the antibiotics listed below, should be considered.		
Ceftazidime-avibactam	Strongly Agree	70/235 (29.8%, 24.3% – 35.7%)
	Agree	123/235 (52.3%, 46% –58.7%)
	Neither Agree or Disagree	39/235 (16.6%, 11.9% – 21.3%)
	Disagree	1/235 (0.4%, 0% – 1.3%)
	Strongly Disagree	2/235 (0.9%, 0% – 2.1%)
Cefiderocol	Strongly Agree	62/235 (26.4%, 20.9% – 32.3%)
	Agree	118/235 (50.2%, 43.8% – 56.6%)
	Neither Agree or Disagree	52/235 (22.1%, 17% – 27.7%)
	Disagree	1/235 (0.4%, 0% – 1.3%)
	Strongly Disagree	2/235 (0.9%, 0% – 2.1%)
Ceftazidime-avibactam Participants working with paediatric patients	Strongly Agree	54/161 (34.8%, 27.4% – 42.3%)
	Agree	84/161 (52.2%, 44.4% – 59.9%)

	Neither Agree or Disagree	18/161 (6.6% – 16.3%)
	Disagree	1/161 (0% – 1.9%)
	Strongly Disagree	2/161 (0% – 3.2%)
Cefiderocol<br>Participants working with paediatric patients.	Strongly Agree	49/161 (30.4%, 23.4% – 37.8%)
	Agree	81/161 (50.3%, 42.7% - 58.1%)
	Neither Agree or Disagree	28/161 (17.5%, 11.7% – 23.3%)
	Disagree	1/161 (0.6%, 0% – 1.9%)
	Strongly Disagree	2/161 (1.2%, 0 – 3.2%)
Ceftazidime-avibactam<br>Participants working with paediatric patients only	Strongly Agree	8/11 (72.7%, 42.9% – 100%)
	Agree	3/11 (27.3%, 0% – 57.1%)
	Neither Agree or Disagree	0%
	Disagree	0%
	Strongly Disagree	0%
Cefiderocol<br>Participants working with paediatric patients only	Strongly Agree	7/11 (63.6%, 33.2% – 90.9%)
	Agree	2/11 (18.2%, 0% – 44.4%)
	Neither Agree or Disagree	2/11 (18.2%, 0% – 44.4%)
	Disagree	0%
	Strongly Disagree	0%

Question 30: The “subscription-type” payment model for antimicrobial drugs requires that individual hospital Trusts still pay a heavily discounted list price for new antimicrobials introduced through the model according to volumes used, while the NHS tops up the remaining difference to the pre-agreed subscription amount.

Under this model, what would you consider to be a fair price for a 24-hour course of ceftazidime/avibactam and cefiderocol, striking the right balance between low enough price to be affordable where clinically indicated and high enough to avoid unjustified use. For comparison, the current cost of a 24-hour course of three times a day standard dose (1g) meropenem in the NHS is £61.14 (BNF, Aug 2022).

Ceftazidime-avibactam (N = 218)	Mean: £193.8 (95% CI 175.6 – 213.5)<br>Median: £150 (IQR 100 – 250)
Cefiderocol (N = 218)	Mean: £231.1 (95% CI 206.9 – 257.1)<br>Median: £200 (IQR 100 – 300)
Ceftazidime-avibactam (N = 214)<br>(Participants with conflicts of interest excluded)	Mean: £193.2 (95% CI 174.1 – 212.2)<br>Median: £150 (IQR 100 – 250)
Cefiderocol (N = 214)<br>(Participants with conflicts of interest excluded)	Mean: £229.5 (95% CI 204.6 – 254.2)<br>Median: £200 (IQR 100 – 300)

Question 31: The ‘subscription-type’ payment model will lead to reduced use of carbapenems.

Strongly Agree	6/235 (2.6%, 0.9% – 4.7%)
Agree	44/235 (18.7%, 14% – 23.8%)
Neither Agree or Disagree	78/235 (33.2%, 27.2% – 39.1%)
Disagree	96/235 (40.9%, 34.5% – 47.2%)
Strongly Disagree	11/235 (4.7%, 2.1 – 7.7%)
Question 32: Outcomes of patients receiving antimicrobials through the 'subscription-type' payment model are being monitored through the UK Antimicrobial Registry, currently being developed by the British Society of Antimicrobial Chemotherapy. Registration of patients is currently voluntary.<br>A national registry is necessary to monitor outcomes of patients, who receive antimicrobials through the 'subscription-type' payment model.	
Strongly Agree	63/235 (26.8%, 21.3% – 32.3%)
Agree	134/235 (57%, 50.6% – 63.4%)
Neither Agree or Disagree	23/235 (9.8%, 6.4% – 13.6%)
Disagree	12/235 (5.1%, 2.6 – 8.1%)
Strongly Disagree	3/235 (1.3%, 0% – 3%)
Question 33: Prescribing indications for antimicrobials introduced through the 'subscription-type' payment model is being monitored through the use of Blueteq forms, which is currently voluntary.<br>Completion of a prescribing authorization process (such as a Blueteq form) during drug dispensing is necessary for regulating the prescribing of antimicrobials provided through the 'subscription-type' payment model.	
Strongly Agree	34/235 (14.8%, 10.2% – 19.1%)
Agree	131/235 (55.7%, 49.4% – 62.1%)
Neither Agree or Disagree	40/235 (17%, 12.3% – 21.7%)
Disagree	25/235 (10.6%, 6.8% –14.9%)
Strongly Disagree	5/235 (2.1%, 0.4% – 4.3%)
Question 34: Completion of a prescribing authorization process (such as a Blueteq form) during dispensing of antimicrobials introduced through the 'subscription-type' payment model should be the responsibility of which of the following professionals (select all that apply)?	
Infectious Diseases or Microbiology Consultant	122/235 (51.9%, 45.6% – 58.2%)
Infectious Diseases or Microbiology Registrar	80/235 (34%, 28.3% – 40.3%)
Antimicrobial stewardship pharmacist	170/235 (72.3%, 66.7% – 78.1%)
Ward pharmacist	70/235 (29.8%, 24.3% – 35.9%)
Clinical team in charge of patient's care	88/235 (37.5%, 31.5% – 43.8%)
Other	5/235 (2.1%, 0.4% – 4.3%)
Completion of a prescribing authorization process should not be necessary	10/235 (4.3%, 2.3% – 7.7%)
Question 35: In your opinion, please select the minimum pre-authorization requirement for the prescription of antimicrobials introduced through the 'subscription-type' payment model.<br>Antimicrobials introduced through the 'subscription-type' payment model can be prescribed:	
By the clinical teams, without consultation from an infection specialist.	3/235 (1.3%, 0% – 3%)
After recommendation from the infectious diseases or microbiology registrar, without the	23/235 (9.8%, 6% – 13.6%)

opinion of the infectious diseases or microbiology consultant.	
After recommendation from a single infectious diseases or microbiology consultant, without the opinion of an antimicrobial stewardship pharmacist.	92/235 (39.2%, 32.8% – 45.5%)
After recommendation from a single infectious diseases or microbiology consultant and a single antimicrobial stewardship pharmacist.	76/235 (32.3%, 26.4% – 38.3%)
After discussion in a multidisciplinary meeting, involving consensus of multiple infectious diseases or microbiology consultants and an antimicrobial stewardship pharmacist.	41/235 (17.5%, 12.8% – 22.6%)
Question 36: In your opinion, antimicrobials introduced through the 'subscription-type' payment model should be available as a treatment option for which of following hierarchical scenarios?	
Empirically, when there is urgent clinical need to treat an unwell patient with a severe infection, in the absence of risk factors for antimicrobial resistance, and all the scenarios below.	2/235 (0.9%, 0% – 2.1%)
Empirically, when there is urgent clinical need to treat an unwell patient with a severe infection, when risk factors for antimicrobial resistance are present, and all the scenarios below.	97/235 (41.3%, 34.9% – 47.7%)
In microbiologically confirmed carbapenem-resistant infections, when microbiological susceptibility and/or genetic testing for the proposed antimicrobial is unknown, and the scenario below	83/235 (35.3%, 29.4% – 41.3%)
In microbiologically confirmed carbapenem-resistant infections, when microbiological susceptibility and/or genetic testing has confirmed that the infection is susceptible to the proposed antimicrobial.	53/235 (22.6%, 17.4% – 28.1%)
Question 37: In your opinion, which of the following scenarios would justify empirical use of antimicrobials introduced through the 'subscription-type' payment model in an unwell patient with a severe infection (select all that apply)?	
Current colonization with carbapenem-resistant bacteria	176/235 (74.9%, 69.4% – 80.4%)
Previous infection with carbapenem-resistant bacteria	190/235 (80.9%, 75.7% – 85.5%)
Clinical treatment failure of broad-spectrum non-carbapenem antibiotics	29/235 (12.3%, 8.1% – 16.6%)
Clinical treatment failure of carbapenem antibiotics	150/235 (63.8%, 57.9% – 69.8%)
Ward outbreak of carbapenem-resistant bacteria	150/235 (63.8%, 57.5% – 69.8%)
Recent admission to a high prevalence hospital for carbapenem-resistant bacteria (in the UK or abroad)	102/235 (43.4%, 37% – 49.8%)

Significant prior broad-spectrum antibiotic exposure (excluding carbapenems)	7/235 (3%, 0.9% –5.1%)
Significant prior exposure to carbapenems	45/235 (19.2%, 14.5% – 24.3%)
Significant prior healthcare exposure (e.g. multiple recent admissions with prolonged length of stay in any hospital)	16/235 (6.8%, 3.8% – 10.2%)
Recent surgery or invasive procedure	3/235 (1.3%, 0% – 3%)
Current or recent admission to Intensive Care	9/235 (3.8%, 1.7% – 6.4%)
None of the above	9/235 (3.8%, 1.7% – 6.4%)
Question 38: In patients with stage IV (severe) or V (end-stage) chronic kidney disease and infections caused by carbapenem-resistant bacteria, ceftazidime/avibactam or cefiderocol are preferable to colistin or aminoglycosides as initial treatment options due to the risks of nephrotoxicity, despite the antimicrobial stewardship need to restrict the use of antimicrobials introduced through the 'subscription-type' payment model.	
Strongly Agree	45/235 (19.2%, 14.5% – 24.3%)
Agree	145 (61.7%, 55.7% – 68.1%)
Neither Agree or Disagree	32/235 (13.6%, 9.4% – 17.9%)
Disagree	9/235 (3.8%, 1.7% – 6.4%)
Strongly Disagree	4/235 (1.7%, 0.4% – 3.4%)
Question 39: In your opinion, please indicate how effective the following interventions are for the antimicrobial stewardship of antimicrobials introduced through the 'subscription-type' payment model in a scale of 1 – 5 (1 = Minimally effective, 5 = Very effective).	
Education of infection specialists	4.07 (3.94 – 4.19)
Education of clinical teams (outside of infection services)	3.31 (3.17 – 3.45)
Local guidelines on patient eligibility criteria	3.93 (3.81 – 4.04)
National guidelines on patient eligibility criteria	3.83 (3.71 – 3.96)
Local audit cycle of appropriateness of prescribing	3.59 (3.46 – 3.71)
Antimicrobial stewardship ward-rounds	4.10 (3.99 – 4.21)
Electronic prescribing systems with prompts/alerts/reminders	3.69 (3.55 – 3.81)
Electronic prescribing systems with controls/automatic stop orders	3.61 (3.47 – 3.74)
Individualized prescriber feedback	3.63 (3.50 – 3.75)
Requirement for pre-authorization by infection specialist	4.22 (4.11 – 4.31)
The presence of an antimicrobial pharmacy team	4.36 (4.26 – 4.45)
Question 40: In my opinion, infections by carbapenem-resistant bacteria in the next 10 years will:	
Increase in number with an exponential growth rate	49/224 (21.9%, 16.5% – 27.7%)
Increase in number with a steady growth rate	158/224 (70.5%, 64.3% – 76.3%)
Increase in number with a damped growth rate	14/224 (6.3%, 3.1% – 9.8%)
Remain the same in number	3/224 (1.3%, 0% – 3.1%)

Decrease in number	0/224 (0%)
Question 41: In vitro susceptibility testing results predict clinical outcomes of infections.	
Strongly Agree	22/235 (9.4%, 6% – 13.2%)
Agree	150/235 (63.8%, 57.9% – 69.8%)
Neither Agree or Disagree	50/235 (21.3%, 16.2% – 26.8%)
Disagree	12/235 (5.1%, 2.6% – 8.1%)
Strongly Disagree	1/235 (0.4%, 0% –1.3%)
Question 42: Susceptibility testing for antimicrobials introduced through the ‘subscription-type’ payment model should be performed (select the best available answer):	
Routinely, for all clinical isolates	19/235 (8.1%, 4.7% – 11.5%)
Routinely, for all clinical isolates, that demonstrate a specific resistance pattern (i.e. ESBL) to first-line agents	81/235 (34.5%, 28.5% – 40.9%)
In special circumstances, where a clinical isolate displays an extensive resistance pattern to routine second-line antimicrobials, and treatment with antimicrobials introduced through the ‘subscription-type’ payment model is considered.	135/235 (57.4%, 51.1% – 63.8%)
Question 43: Isolates resistant to antimicrobials introduced through the ‘subscription-type’ payment model should be sent to a reference laboratory for further characterization.	
Strongly Agree	60/235 (25.5%, 20% – 31.1%)
Agree	109/235 (46.4%, 40% – 52.8%)
Neither Agree or Disagree	44/235 (18.7%, 14% – 23.8%)
Disagree	20/235 (8.5%, 5.1 – 12.3%)
Strongly Disagree	2/235 (0.9%, 0% – 2.1%)
Question 44: How does your hospital laboratory perform susceptibility testing for ceftazidime/avibactam (select all that apply)?	
In-house Broth microdilution assay	22/235 (9.4%)
In-house disc diffusion	59 (25.1%)
In-house Etest	122/235 (51.9%)
Reference laboratory	23/235 (9.8%)
My hospital does not perform susceptibility testing for ceftazidime/avibactam	5/235 (2.1%)
Other (please specify)	7/235 (3%)
I do not know	55/235 (23.4%)
Question 45: How does your hospital laboratory perform susceptibility testing for cefiderocol (select all that apply)?	
In-house Broth microdilution assay	19/235 (8.1%)
In-house disc diffusion	92/235 (39.2%)
Reference laboratory	50/235 (21.3%)
My hospital does not perform susceptibility testing for ceftazidime/avibactam	22/235 (9.4%)
Other (please specify)	7/235 (3%)
I do not know	74/235 (31.5%)

**Question 46: How does your hospital laboratory perform testing for the detection of acquired carbapenemases (select all that apply)?**	
Nucleic acid amplification test (real-time PCR)	129/235 (54.9%)
Immunochromatographic assay	53/235 (22.6%)
Syndromic assays (PCR and DNA hybridization)	5/235 (2.1%)
Reference laboratory	51/235 (21.7%)
My hospital laboratory does not perform testing for the detection of acquired carbapenemases.	0/235 (0%)
Other (please specify)	19/235 (8.1%)
I do not know	43/235 (18.3%)
**Question 47: At the launch of the 'subscription-type' payment model I had received adequate information about it.**	
Strongly Agree	13/235 (5.5%, 2.6% – 8.5%)
Agree	25/235 (10.6%, 6.8% – 14.9%)
Neither Agree or Disagree	47/235 (20%, 14.9% – 25.1%)
Disagree	108/235 (46%, 39.6% – 52.3%)
Strongly Disagree	42/235 (17.9%, 13.2 – 23%)
**Question 48: In your opinion, please indicate how effective the following methods are as learning methods on the use of novel antimicrobials in a scale of 1 – 5 (1 = Minimally effective, 5 = Very effective).**	
Informal on-the-job teaching	3.51 (3.39 – 3.63)
Formal organized teaching sessions in the workplace	3.86 (3.76 – 3.95)
Externally organized scientific meetings (conferences, workshops, webinars etc.)	3.81 (3.70 – 3.92)
Scientific publications	3.54 (3.42 – 3.66)
Guidance published by NHSE, NICE or UKHSA	4.02 (3.91 – 4.13)
Other (please specify)	No responses
**Question 49: Would you like to receive the written report of this survey?**	
Yes	156/235 (66.4%)
No	79/235 (33.6%)
**Question 50: To test the reliability of the SMASH questionnaire tool, we would like a small subset of participants to repeat the survey approximately one month after their original submission. A second £10 Amazon voucher will be provided for this effort. Please indicate below if you would like to be invited to repeat the survey.**	
Yes, I would be willing to repeat the SMASH survey	133/235 (56.6%)
No, thank you, I would not be willing to repeat the SMASH survey	102/235 (43.4%)
**Question 51: Please provide your NHS email address below. This will only be used to send you your £10 Amazon voucher (in January 2023), invite you to repeat the survey if you have indicated you would be willing to so do, and send you the survey results, if requested. Your email address will not be used for any other purposes including sending you spam emails, will not be shared with the funder of this study and will not be stored after the end of this study.**	
Responses to this question were not published.	

NHS: National Health Service; CI: confidence interval; IQR: interquartile range
95% CIs were calculated using 10.000 bootstrap samples

Supplementary table 2	
Question	Cohen's kappa
1	Not performed**
2	Not performed**
3	Not performed**
4	Not performed**
5	Not performed**
6	Not performed**
7	Not performed**
8	Not performed**
9	Not performed**
10	0.64*
11	0.66*
12	0.68*
13	0.31*
14	0.46*
15	0.60*
16	Not performed**
17	Not performed**
18	Not performed**
19	Not performed**
20	Not performed**
21 - Meropenem	0.48*
21 - Ceftazidime-avibactam	0.60*
21 - Cefiderocol	0.73*
22	Not performed**
23	Not performed**
24	Not performed**
25	Not performed**
26	Not performed**
27	0.45*
28	0.61*
29 – Ceftazidime-avibactam	0.49*
29 - Cefiderocol	0.55*
30	Not performed**
31	0.36*
32	0.49*
33	0.57*
34	Not performed**
35	0.35

36	0.37
37 - Current colonization with carbapenem-resistant bacteria	0.28
37 - Previous infection with carbapenem-resistant bacteria	0.43
37 - Clinical treatment failure of broad-spectrum non-carbapenem antibiotics	NA
37 - Clinical treatment failure of carbapenem antibiotics	0.57
37 - Ward outbreak of carbapenem-resistant bacteria	0.24
37 - Recent admission to a high prevalence hospital for carbapenem-resistant bacteria (in England or abroad)	0.36
37 - Significant prior broad-spectrum antibiotic exposure (excluding carbapenems)	NA
37 - Significant prior exposure to carbapenems	0.37
37 - Significant prior healthcare exposure (e.g. multiple recent admissions with prolonged length of stay)	NA
37 - Recent surgery or invasive procedure	NA
37 - Current or recent admission to Intensive Care	NA
37 - None of the above	0.65
38	0.46*
39	Not performed**
40	0.55*
41	0.65*
42	0.41*
43	0.212*
44	Not performed**
45	Not performed**
46	Not performed**
47	0.64 (0.64 – 0.64)*
48	Not performed**
49	Not performed**
50	Not performed**
51	Not performed**

*Denotes weighted kappa, **Analysis not performed either because the variable was continuous or because of the nature of the question (response likely to change with time or fixed response). NA: not applicable

Supplementary Table 3: Cronbach's alpha calculations	
Questions	a
10, 11, 15	0.732
21, 22, 23	0.841
27, 28, 38	0.623
32, 33	0.708

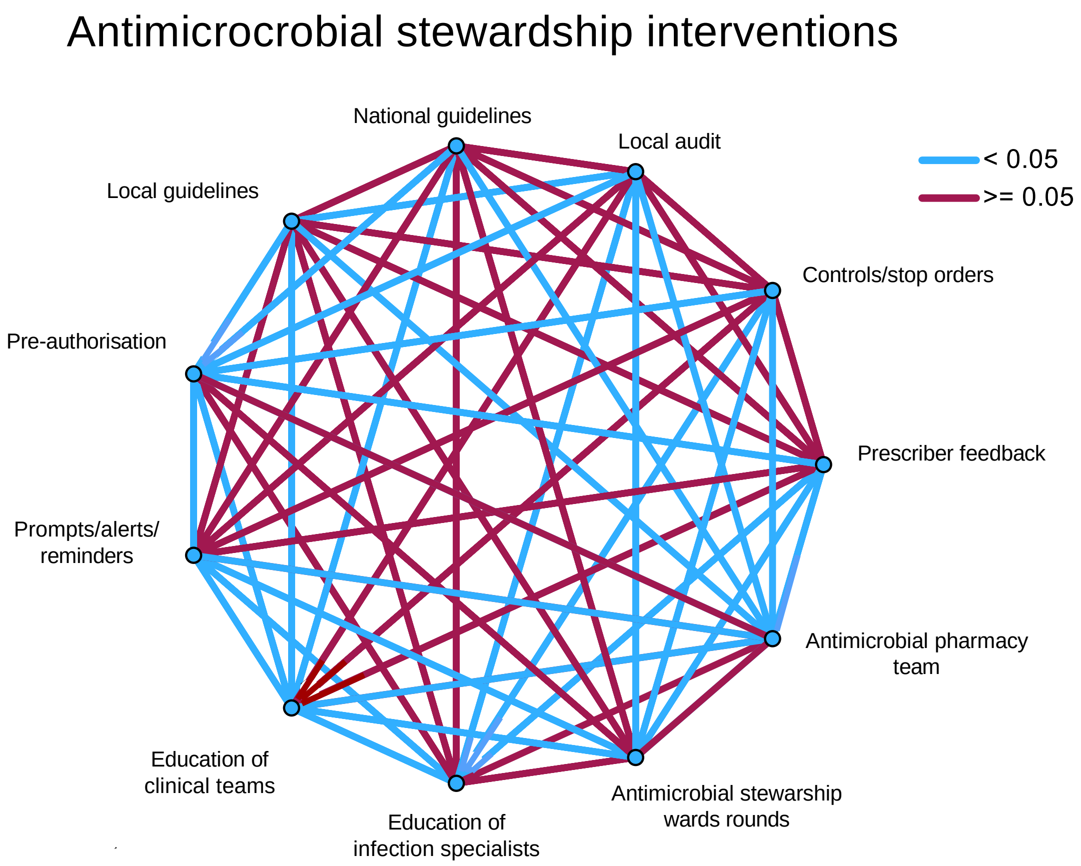

The presence of an antimicrobial pharmacy team	4.36 (4.26 – 4.45)
Requirement for pre-authorization by infection specialist	4.22 (4.11 – 4.31)
Antimicrobial stewardship ward-rounds	4.10 (3.99 – 4.21)
Education of infection specialists	4.07 (3.94 – 4.19)
Local guidelines on patient eligibility criteria	3.93 (3.81 – 4.04)
National guidelines on patient eligibility criteria	3.83 (3.71 – 3.96)
Electronic prescribing systems with prompts/alerts/reminders	3.69 (3.55 – 3.81)
Individualized prescriber feedback	3.63 (3.50 – 3.75)
Electronic prescribing systems with controls/automatic stop orders	3.61 (3.47 – 3.74)
Local audit cycle of appropriateness of prescribing	3.59 (3.46 – 3.71)
Education of clinical teams (outside of infection services)	3.31 (3.17 – 3.45)

Supplementary Figure 1: Effectiveness of antimicrobial stewardship interventions. Pairwise comparisons (Kruskal Wallis test), means and 95% CIs of values given by participants to the effectiveness of eleven antimicrobial stewardship interventions using a 5-point numerical scale (1 = Minimally effective, 5 = Very effective). Red lines connect interventions without a statistically significant pairwise comparison ($p \geq 0.05$), while blue lines connect interventions with a statistically significant pairwise comparison ($p<0.05$). All pairwise comparisons are adjusted using the Bonferroni correction. 95% CIs were calculated using 10.000 bootstrap samples.

LONDON SCHOOL of HYGIENE &TROPICAL MEDICINE

SMASH

SUBSCRIPTION MODELS FOR ANTIBIOTICS IN HOSPITALS

SMASH survey

Welcome to the SMASH survey (Subscription Models for AntibioticS in Hospitals).

Dear Colleague,

Thank you for considering completing the SMASH survey (Subscription Models for AntibioticS in Hospitals). This study is being conducted by researchers at the London School of Hygiene and Tropical Medicine and was designed in consultation with NHS England. It is an opportunity for you to express your views on the implementation of a novel funding model for antimicrobials in the National Health Service (NHS). We hope that the findings of this survey will influence future health policy and improve the care of patients with drug-resistant infections in England.

The SMASH survey should take approximately 20 minutes to complete. To thank you for your time in completing this survey, you will be provided with a £10 Amazon voucher (to be sent electronically in January 2023).

Study background:

On 1st July, 2022 NHS England, in collaboration with the National Institute for Health and Care Excellence (NICE), launched a novel “subscription-type” payment model for two new antimicrobials, ceftazidime/avibactam and cefiderocol. This de-linked funding model means that the NHS will pay the pharmaceutical companies producing these drugs a fixed pre-agreed annual fee (the “subscription”), irrespective of volumes of antimicrobials actually used by individual Trusts. So, rather than paying for these two drugs on a “number of vials used” basis, this funding model can be considered as a “subscription for access” for these two agents for the NHS as a whole. The individual NHS Trusts still pay a discounted list price for these drugs – NHS England tops up these payments received to reach the undisclosed pre-agreed subscription amount (up to a maximum of £10million/year). This funding model is intended to allow affordable use of these two antimicrobials for patients with drug-resistant infections in individual Trusts. It also aims to align industry financial incentives with good stewardship practices, and incentivise pharmaceutical companies to bring new antimicrobial products to market. This initiative is part of the 2019 - 2024 UK Antimicrobial Resistance 5-year National Action Plan and represents the world’s first fully delinked funding model for antimicrobials. The length of the initial agreement contract for this model is 3 years, with an option to extend up to 10 years.

The two antibiotics currently included in the subscription model are ceftazidime/avibactam (produced by Pfizer) and cefiderocol (produced by Shionogi), which are licenced for treating severe drug-resistant infections caused by Gram-negative bacteria. As part of the appraisal process for this new funding model, the prescribing indications for these antibiotics are being monitored using Blueteq forms, and patient outcomes are being monitored through the UK Antimicrobial Registry, currently being developed by the British Society of Antimicrobial Chemotherapy. Both monitoring modalities are currently voluntary. This survey collects complementary information to these other modalities of evaluation. You can find additional information on the new subscription-type payment model in the following link:

https://www.nice.org.uk/about/what-we-do/life-sciences/scientific-advice/models-for-the-evaluation-and-purchase-of-antimicrobials

The SMASH survey aims to capture the views of Infectious Diseases and Microbiology Consultants on the implementation and impact of the novel subscription-type payment model in the NHS. This should allow us to collate expert opinion on important aspects of the de-linked funding models, as well as inform upcoming contract negotiations and future health policy. The SMASH questionnaire is not a test of knowledge and aims to capture participant opinions only. This study is conducted by the London School of Hygiene and Tropical Medicine (REC Reference No 28161/RR/29296) and is funded by an independent research grant from Shionogi B.V. The funder of this study has had/will have no role in the design, conduct or interpretation of this study, nor will details of study participants be shared with the funder. Survey results will be pooled and analyzed at regional level, while individual responses will be stored in password protected NHS computers, to preserve anonymity and confidentiality of study participants. At the end of the survey, you will be invited to indicate whether you would be interested to participate in a second round of data collection.

Thank you for your consideration and your time,

Dr Ioannis Baltas,[1] Mr Mark Gilchrist[2] & Dr Alexander Aiken,[3]
On behalf of the SMASH team

[1]declares funding for conducting this survey and honoraria from Shionogi B.V.

[2]declares being a member of the Advisory Board for Pfizer.

[3]declares no conflicts of interest beyond the funding received for conduct of this survey from Shionogi B.V.

By clicking the “Next” button below, you consent to participate in this study.

LONDON SCHOOL of HYGIENE &TROPICAL MEDICINE

SMASH

SUBSCRIPTION MODELS FOR ANTIBIOTICS IN HOSPITALS

SMASH survey

Survey Eligibility

* 1. Are you a Consultant in either Infectious Diseases, Medical Microbiology, Infectious Diseases and Medical Microbiology, or Infectious Diseases and General Internal Medicine currently working at the least 2 Programmed activities (PAs) per week or equivalent in a substantive or locum post of an NHS acute hospital?

- ◯ Yes
- ◯ No

LONDON SCHOOL of HYGIENE &TROPICAL MEDICINE

SMASH

SUBSCRIPTION MODELS FOR ANTIBIOTICS IN HOSPITALS

SMASH survey

Demographics

* 2. Please select the specialty/combination of specialties that you currently hold a Certificate of completion of training (CCT) or Certificate of completion of Specialty training (CCST) from the General Medical Council (GMC), or equivalent in country of specialist training:

- ○ Infectious Diseases
- ○ Medical Microbiology
- ○ Infectious Diseases and Medical Microbiology
- ○ Infectious Diseases and General Internal Medicine

* 3. Please state the year that you received the above CCT/CCST (or overseas equivalent):

[ ]

* 4. Please indicate the NHS region you are currently working in:

- ○ East of England
- ○ London
- ○ Midlands
- ○ North East and Yorkshire
- ○ North West
- ○ South East
- ○ South West

* 5. Please provide the name of the hospital/hospitals you currently work in (this will only be used to assess survey sample representativeness and not to identify you, please avoid abbreviations):

[ ]

* 6. Please indicate the type of patients you currently work with:

- ◯ Adult
- ◯ Paediatric
- ◯ Both

* 7. Do you have any relevant conflicts of interest when completing this survey (this does not preclude you from completing the survey)?

- ◯ Yes
- ◯ No

LONDON SCHOOL of HYGIENE &TROPICAL MEDICINE

SMASH

SUBSCRIPTION MODELS FOR ANTIBIOTICS IN HOSPITALS

SMASH survey

Conficts of interest

8. Please declare your conficts of interest:

LONDON SCHOOL of HYGIENE & TROPICAL MEDICINE

SMASH

SUBSCRIPTION MODELS FOR ANTIBIOTICS IN HOSPITALS

SMASH survey

The antimicrobial subscription model

* 9. Before completing this survey, had you previously heard about the ‘subscription-type’ payment model for antimicrobial drugs?

- ◯ Yes
- ◯ No

On the scale shown, how much do you agree with the following statements about the UK’s ‘subscription-type’ payment model for antimicrobial drugs?

* 10. The ‘subscription-type’ payment model is a welcome development in the management of drug-resistant infections.

- ◯ Strongly agree
- ◯ Agree
- ◯ Neither agree nor disagree
- ◯ Disagree
- ◯ Strongly disagree

* 11. Access to novel antimicrobials through the ‘subscription-type’ payment model will improve the ability of infection specialists to treat drug-resistant infections.

- ◯ Strongly agree
- ◯ Agree
- ◯ Neither agree nor disagree
- ◯ Disagree
- ◯ Strongly disagree

* 12. Cost is a significant consideration for me when using/recommending the use of specific antimicrobials:

- Strongly agree
- Agree
- Neither agree nor disagree
- Disagree
- Strongly disagree

* 13. Access to novel antimicrobials through the 'subscription-type' payment model will improve cost-effectiveness in the management of drug-resistant infections.

- Strongly agree
- Agree
- Neither agree nor disagree
- Disagree
- Strongly disagree

* 14. Prescribing indications for antimicrobials supplied through the 'subscription-type' payment model are being monitored through the use of Blueteq forms, while outcomes of patients are being monitored through the UK Antimicrobial Registry. Both monitoring modalities are currently voluntary.

This will increase your personal administrative workload in the management of drug-resistant infections compared to previous local arrangements (e.g. completion of non-formulary or unlicensed drug requests, requests for financial approval for excess drug acquisition costs) for acquiring these antimicrobials.

- Strongly agree
- Agree
- Neither agree nor disagree
- Disagree
- Strongly disagree

* 15. The 'subscription-type' payment model will stimulate research and development of new antimicrobials.

- Strongly agree
- Agree
- Neither agree nor disagree
- Disagree
- Strongly disagree

SMASH survey

STEDI values (Spectrum, Transmission, Enablement, Diversity, Insurance)

* 16. The term “insurance value” refers to the value of having antibiotics available in case of a sudden or major increase in the prevalence of infections with pathogens resistant to all other existing antibiotics. On a scale of 1 – 5, please assess the insurance value of the following antibiotics:

	1	2	3	4	5	Cannot say
Ceftazidime/avibactam	○	○	○	○	○	○
Cefiderocol	○	○	○	○	○	○
Colistin	○	○	○	○	○	○
Tigecycline	○	○	○	○	○	○
Fosfomycin	○	○	○	○	○	○
Amikacin	○	○	○	○	○	○
Meropenem	○	○	○	○	○	○
Ceftolozane/tazobactam	○	○	○	○	○	○
Meropenem/vaborbactam	○	○	○	○	○	○

1 = Low insurance value 3 = Medium insurance value 5 = High insurance value

* 17. The term “enablement value” refers to the benefits associated with enabling other treatments or procedures to take place, e.g., surgical and medical procedures that may not be possible if antimicrobials were not available to prevent or treat surgical site or post-procedure infections. On a scale of 1 – 5, please assess the enablement value of the following antibiotics, given the current prevalence of resistant infections:

	1	2	3	4	5	Cannot say
Ceftazidime/avibactam	○	○	○	○	○	○
Cefiderocol	○	○	○	○	○	○
Colistin	○	○	○	○	○	○
Tigecycline	○	○	○	○	○	○
Fosfomycin	○	○	○	○	○	○
Amikacin	○	○	○	○	○	○
Meropenem	○	○	○	○	○	○
Ceftolozane/tazobactam	○	○	○	○	○	○
Meropenem/vaborbactam	○	○	○	○	○	○

1 = Low enablement value 3 = Medium enablement value 5 = High enablement value

* 18. The term “transmission value” refers to the benefits of avoiding the spread of the pathogen to the wider population if the patient with the infection responds promptly to treatment and is treated successfully. On a scale of 1 – 5, please assess the transmission value of the following antibiotics:

	1	2	3	4	5	Cannot say
Ceftazidime/avibactam	○	○	○	○	○	○
Cefiderocol	○	○	○	○	○	○
Colistin	○	○	○	○	○	○
Tigecycline	○	○	○	○	○	○
Fosfomycin	○	○	○	○	○	○
Amikacin	○	○	○	○	○	○
Meropenem	○	○	○	○	○	○
Ceftolozane/tazobactam	○	○	○	○	○	○
Meropenem/vaborbactam	○	○	○	○	○	○

1 = Low transmission value 3 = Medium transmission value 5 = High transmission value

* 19. The term “diversity value” refers to the benefits of having a range of treatment options available to reduce selection pressure for resistance and to preserve the efficacy of existing antimicrobials. For example, a new antibiotic, typically effective against Vancomycin-resistant Staphylococcus aureus, where alternative treatment options are limited, would have high diversity value, while a new antibiotic typically effective against Group A streptococcus, where multiple alternative treatment options are available, would have low diversity value. On a scale of 1 – 5, please assess the diversity value of having/adding the following antibiotics to your existing hospital formulary:

	1	2	3	4	5	Cannot say
Ceftazidime/avibactam	◯	◯	◯	◯	◯	◯
Cefiderocol	◯	◯	◯	◯	◯	◯
Colistin	◯	◯	◯	◯	◯	◯
Tigecycline	◯	◯	◯	◯	◯	◯
Fosfomycin	◯	◯	◯	◯	◯	◯
Amikacin	◯	◯	◯	◯	◯	◯
Meropenem	◯	◯	◯	◯	◯	◯
Ceftolozane/tazobactam	◯	◯	◯	◯	◯	◯
Meropenem/vaborbactam	◯	◯	◯	◯	◯	◯

1 = Low diversity value 3 = Medium diversity value 5 = High diversity value

* 20. The term “spectrum value” refers to the benefits of replacing other broad-spectrum antibiotics that could be used to cure the same infection, with a narrower spectrum antibiotic. For example, an antibiotic with a spectrum similar to flucloxacillin would be considered to have high spectrum value, while an antibiotic with a spectrum similar to piperacillin/tazobactam would be considered to have low spectrum value. On a scale of 1 – 5, please assess the spectrum value of the following antibiotics for replacing broad-spectrum agents:

	1	2	3	4	5	Cannot say
Ceftazidime/avibactam	◯	◯	◯	◯	◯	◯
Cefiderocol	◯	◯	◯	◯	◯	◯
Colistin	◯	◯	◯	◯	◯	◯
Tigecycline	◯	◯	◯	◯	◯	◯
Fosfomycin	◯	◯	◯	◯	◯	◯
Amikacin	◯	◯	◯	◯	◯	◯
Meropenem	◯	◯	◯	◯	◯	◯
Ceftolozane/tazobactam	◯	◯	◯	◯	◯	◯
Meropenem/vaborbactam	◯	◯	◯	◯	◯	◯

1 = Low spectrum value 3 = Medium spectrum value 5 = High spectrum value

LONDON SCHOOL of HYGIENE &TROPICAL MEDICINE

SMASH

SUBSCRIPTION MODELS FOR ANTIBIOTICS IN HOSPITALS

SMASH survey

Clinical use and indications for antimicrobials

* 21. Please indicate how confident you feel with recognizing the type of infections that would require treatment with the following antibiotics?

	Not at all confident	Not so confident	Somewhat confident	Very confident	Extremely confident
Meropenem	○	○	○	○	○
Ceftazidime/Avibactam	○	○	○	○	○
Cefiderocol	○	○	○	○	○

* 22. In your routine clinical practice **before** the introduction of the ‘subscription-type’ payment model (ie before 1st July 2022), which of the following options best represents the frequency that you used/ recommended use of the following antibiotics?

	Never	Every 3 - 4 months	Monthly	Weekly	Daily
Meropenem	○	○	○	○	○
Ceftazidime/avibactam	○	○	○	○	○
Cefiderocol	○	○	○	○	○

* 23. In your routine current clinical practice **after** the introduction of the ‘subscription-type’ payment model (ie after 1st July 2022), which of the following options best represents the frequency that you use/ recommend use of the following antibiotics?

	Never	Every 3 - 4 months	Monthly	Weekly	Daily
Meropenem	○	○	○	○	○
Ceftazidime/avibactam	○	○	○	○	○
Cefiderocol	○	○	○	○	○

* 24. In your opinion, the listed antibiotics are an important treatment option for infections caused by which of the following microorganisms (select all that apply):

	Meropenem	Ceftazidime/avibactam	Cefiderocol
Escherichia coli	☐	☐	☐
Klebsiella pneumonia	☐	☐	☐
Klebsiella oxytoca	☐	☐	☐
Pseudomonas aeruginosa	☐	☐	☐
Enterobacter spp.	☐	☐	☐
Citrobacter spp.	☐	☐	☐
Morganella spp.	☐	☐	☐
Serratia spp.	☐	☐	☐
Acinetobacter spp.	☐	☐	☐
Burkholderia cepacia complex	☐	☐	☐
Stenotrophomonas maltophilia	☐	☐	☐

* 25. In your opinion, the listed antibiotics are an important treatment option for infections caused by microorganisms with which of the following resistance mechanisms (select all that apply):

	Meropenem	Ceftazidime/avibactam	Cefiderocol
NDM	☐	☐	☐
KPC	☐	☐	☐
OXA-48	☐	☐	☐
IMP	☐	☐	☐
VIM	☐	☐	☐
Non-carbapenemase carbapenem resistance	☐	☐	☐
ESBL	☐	☐	☐
AmpC	☐	☐	☐

Key - NDM: New Delhi Metallo-beta-lactamase, KPC: Klebsiella pnuemoniae carbapenemase, OXA-48: Oxacillinase-48, IMP: Imipenemase, VIM: Verona integrated-encoded Metallo-beta-lactamase, ESBL: Extended-spectrum β-lactamase, AmpC: Ampicillinase C

* 26. In your opinion, the listed antibiotics are an important treatment option for which of the following sites of infection (select all that apply):

	Meropenem	Ceftazidime/avibactam	Cefiderocol
Bloodstream infection	☐	☐	☐
Pneumonia – hospital acquired	☐	☐	☐
Pneumonia – ventilator associated	☐	☐	☐
Complicated urinary tract infection	☐	☐	☐
Complicated intra-abdominal infection	☐	☐	☐
Complicated skin and soft tissue infection	☐	☐	☐
Bone / joint infection	☐	☐	☐
Central nervous system infection	☐	☐	☐
Cystic fibrosis bronchiectasis	☐	☐	☐

* 27. Ceftazidime/avibactam is associated with less treatment-related toxicity than other locally available antibiotic regimens for the treatment of OXA-48-producing carbapenem-resistant infections.

- ◯ Strongly agree
- ◯ Agree
- ◯ Neither agree nor disagree
- ◯ Disagree
- ◯ Strongly disagree

* 28. Cefiderocol is associated with less treatment-related toxicity than other locally available antibiotic regimens for the treatment of metallo-beta-lactamase-producing carbapenem-resistant infections.

- ◯ Strongly agree
- ◯ Agree
- ◯ Neither agree nor disagree
- ◯ Disagree
- ◯ Strongly disagree

* 29. Off-licence treatment of paediatric patients (<18 years old) with severe infections due to Gram-negative bacteria with limited treatment options, with the antibiotics listed below, should be considered.

	Strongly agree	Agree	Neither agree or disagree	Disagree	Strongly disagree
Ceftazidime/avibactam	○	○	○	○	○
Cefiderocol	○	○	○	○	○

* 30. The “subscription-type” payment model for antimicrobial drugs requires that individual hospital Trusts still pay a heavily discounted list price for new antimicrobials introduced through the model according to volumes used, while the NHS tops up the remaining difference to the pre-agreed subscription amount.

Under this model, what would you consider to be a fair price for a 24-hour course of ceftazidime/avibactam and cefiderocol, striking the right balance between low enough price to be affordable where clinically indicated and high enough to avoid unjustified use. For comparison, the current cost of a 24-hour course of three times a day standard dose (1g) meropenem in the NHS is £61.14 (BNF, Aug 2022).

Ceftazidime/avibactam [ ]

Cefiderocol [ ]

SMASH survey

Antimicrobial stewardship

On the scale shown, how much do you agree with the following statements about the antimicrobial stewardship of the antimicrobials introduced through the ‘subscription-type’ payment model?

* 31. The ‘subscription-type’ payment model will lead to reduced use of carbapenems.

- Strongly agree
- Agree
- Neither agree nor disagree
- Disagree
- Strongly disagree

* 32. Outcomes of patients receiving antimicrobials through the ‘subscription-type’ payment model are being monitored through the UK Antimicrobial Registry, currently being developed by the British Society of Antimicrobial Chemotherapy. Registration of patients is currently voluntary.

A national registry is necessary to monitor outcomes of patients, who receive antimicrobials through the ‘subscription-type’ payment model.

- Strongly agree
- Agree
- Neither agree nor disagree
- Disagree
- Strongly disagree

* 33. Prescribing indications for antimicrobials introduced through the ‘subscription-type’ payment model is being monitored through the use of Blueteq forms, which is currently voluntary.

Completion of a prescribing authorization process (such as a Blueteq form) during drug dispensing is necessary for regulating the prescribing of antimicrobials provided through the ‘subscription-type’ payment model.

- ◯ Strongly agree
- ◯ Agree
- ◯ Neither agree nor disagree
- ◯ Disagree
- ◯ Strongly disagree

* 34. Completion of a prescribing authorization process (such as a Blueteq form) during dispensing of antimicrobials introduced through the ‘subscription-type’ payment model should be the responsibility of which of the following professionals (select all that apply)?

- ☐ Infectious Diseases or Microbiology Consultant
- ☐ Infectious Diseases or Microbiology Registrar
- ☐ Antimicrobial stewardship pharmacist
- ☐ Ward pharmacist
- ☐ Clinical team in charge of patient’s care
- ☐ Other (please specify)

  ____

- ☐ Completion of a prescribing authorization process should not be necessary

* 35. In your opinion, please select the **minimum** pre-authorization requirement for the prescription of antimicrobials introduced through the ‘subscription-type’ payment model.

Antimicrobials introduced through the ‘subscription-type’ payment model can be prescribed:

- ◯ By the clinical teams, without consultation from an infection specialist.
- ◯ After recommendation from the infectious diseases or microbiology registrar, without the opinion of the infectious diseases or microbiology consultant.
- ◯ After recommendation from a single infectious diseases or microbiology consultant, without the opinion of an antimicrobial stewardship pharmacist.
- ◯ After recommendation from a single infectious diseases or microbiology consultant and a single antimicrobial stewardship pharmacist.
- ◯ After discussion in a multidisciplinary meeting, involving consensus of multiple infectious diseases or microbiology consultants and an antimicrobial stewardship pharmacist.

* 36. In your opinion, antimicrobials introduced through the ‘subscription-type’ payment model should be available as a treatment option for which of following hierarchical scenarios?

- ◯ Empirically, when there is urgent clinical need to treat an unwell patient with a severe infection, in the absence of risk factors for antimicrobial resistance, and all the scenarios below.
- ◯ Empirically, when there is urgent clinical need to treat an unwell patient with a severe infection, when risk factors for antimicrobial resistance are present, and all the scenarios below.
- ◯ In microbiologically confirmed carbapenem-resistant infections, when microbiological susceptibility and/or genetic testing for the proposed antimicrobial is unknown, and the scenario below.
- ◯ In microbiologically confirmed carbapenem-resistant infections, when microbiological susceptibility and/or genetic testing has confirmed that the infection is susceptible to the proposed antimicrobial.

* 37. In your opinion, which of the following scenarios would justify empirical use of antimicrobials introduced through the ‘subscription-type’ payment model in an unwell patient with a severe infection (select all that apply)?

- ☐ Current colonization with carbapenem-resistant bacteria
- ☐ Previous infection with carbapenem-resistant bacteria
- ☐ Clinical treatment failure of broad-spectrum non-carbapenem antibiotics
- ☐ Clinical treatment failure of carbapenem antibiotics
- ☐ Ward outbreak of carbapenem-resistant bacteria
- ☐ Recent admission to a high prevalence hospital for carbapenem-resistant bacteria (in England or abroad)
- ☐ Significant prior broad-spectrum antibiotic exposure (excluding carbapenems)
- ☐ Significant prior exposure to carbapenems
- ☐ Significant prior healthcare exposure (e.g. multiple recent admissions with prolonged length of stay)
- ☐ Recent surgery or invasive procedure
- ☐ Current or recent admission to Intensive Care
- ☐ None of the above

* 38. In patients with stage IV (severe) or V (end-stage) chronic kidney disease and infections caused by carbapenem-resistant bacteria, ceftazidime/avibactam or cefiderocol are preferable to colistin or aminoglycosides as initial treatment options due to the risks of nephrotoxicity, despite the antimicrobial stewardship need to restrict the use of antimicrobials introduced through the ‘subscription-type’ payment model.

- ◯ Strongly agree
- ◯ Agree
- ◯ Neither agree nor disagree
- ◯ Disagree
- ◯ Strongly disagree

* 39. In your opinion, please indicate how effective the following interventions are for the antimicrobial stewardship of antimicrobials introduced through the ‘subscription-type’ payment model in a scale of 1 – 5 (1 = Minimally effective, 5 = Very effective)

	1	2	3	4	5
Education of infection specialists	◯	◯	◯	◯	◯
Education of clinical teams (outside of infection services)	◯	◯	◯	◯	◯
Local guidelines on patient eligibility criteria	◯	◯	◯	◯	◯
National guidelines on patient eligibility criteria	◯	◯	◯	◯	◯
Local audit cycle of appropriateness of prescribing	◯	◯	◯	◯	◯
Antimicrobial stewardship ward-rounds	◯	◯	◯	◯	◯
Electronic prescribing systems with prompts/alerts/reminders	◯	◯	◯	◯	◯
Electronic prescribing systems with controls/automatic stop orders	◯	◯	◯	◯	◯
Individualized prescriber feedback	◯	◯	◯	◯	◯
Requirement for pre-authorization by infection specialist	◯	◯	◯	◯	◯
The presence of an antimicrobial pharmacy team	◯	◯	◯	◯	◯

The following question is **not mandatory**. Please complete it only if you wish to.

40. In my opinion, infections by carbapenem-resistant bacteria in the next 10 years will:

- ◯ Increase in number with an exponential growth rate
- ◯ Increase in number with a steady growth rate
- ◯ Increase in number with a damped growth rate
- ◯ Remain the same in number
- ◯ Decrease in number

LONDON SCHOOL of HYGIENE &TROPICAL MEDICINE

SMASH

SUBSCRIPTION MODELS FOR ANTIBIOTICS IN HOSPITALS

SMASH survey

Antimicrobial resistance testing

On the scale shown, how much do you agree with the following statements about susceptibility testing of the antimicrobials introduced through the ‘subscription-type’ payment model?

* 41. In vitro susceptibility testing results predict clinical outcomes of infections.

- ◯ Strongly agree
- ◯ Agree
- ◯ Neither agree nor disagree
- ◯ Disagree
- ◯ Strongly disagree

* 42. Susceptibility testing for antimicrobials introduced through the ‘subscription-type’ payment model should be performed (select the best available answer):

- ◯ Routinely, for all clinical isolates
- ◯ Routinely, for all clinical isolates, that demonstrate a specific resistance pattern (i.e. ESBL) to first-line agents
- ◯ In special circumstances, where a clinical isolate displays an extensive resistance pattern to routine second-line antimicrobials, and treatment with antimicrobials introduced through the ‘subscription-type’ payment model is considered.

* 43. Isolates resistant to antimicrobials introduced through the ‘subscription-type’ payment model should be sent to a reference laboratory for further characterization.

- ◯ Strongly agree
- ◯ Agree
- ◯ Neither agree nor disagree
- ◯ Disagree
- ◯ Strongly disagree

* 44. How does your hospital laboratory perform susceptibility testing for ceftazidime/avibactam (select all that apply)?

- [ ] In-house Broth microdilution assay
- [ ] In-house disc diffusion
- [ ] In-house Etest
- [ ] Reference laboratory
- [ ] I do not know
- [ ] Other (please specify)
- [ ] My hospital laboratory does not perform susceptibility testing for ceftazidime/avibactam.

* 45. How does your hospital laboratory perform susceptibility testing for cefiderocol (select all that apply)?

- [ ] In-house Broth microdilution assay
- [ ] In-house disc diffusion
- [ ] Reference laboratory
- [ ] I do not know
- [ ] Other (please specify)
- [ ] My hospital laboratory does not perform susceptibility testing for cefiderocol.

46. How does your hospital laboratory perform testing for the detection of acquired carbapenemases (select all that apply)?

- [ ] Nucleic acid amplification test (real-time PCR)
- [ ] Immunochromatographic assay
- [ ] Syndromic assays (PCR and DNA hybridization)
- [ ] Reference laboratory
- [ ] I do not know
- [ ] Other (please specify)
- [ ] My hospital laboratory does not perform testing for the detection of acquired carbapenemases.

LONDON SCHOOL of HYGIENE &TROPICAL MEDICINE

SMASH

SUBSCRIPTION MODELS FOR ANTIBIOTICS IN HOSPITALS

SMASH survey

Teaching and learning

* 47. At the launch of the 'subscription-type' payment model I had received adequate information about it.

- Strongly agree
- Agree
- Neither agree nor disagree
- Disagree
- Strongly disagree

* 48. In your opinion, please indicate how effective the following methods are as learning methods on the use of novel antimicrobials in a scale of 1 – 5 (1 = Minimally effective, 5 = Very effective)

	1	2	3	4	5
Informal on-the-job teaching	○	○	○	○	○
Formal organized teaching sessions in the workplace	○	○	○	○	○
Externally organized scientific meetings (conferences, workshops, webinars etc.)	○	○	○	○	○
Scientific publications	○	○	○	○	○
Guidance published by NHSE, NICE or UKHSA	○	○	○	○	○

Other (please specify)

1 = Minimally effective, 10 = Very effective

LONDON SCHOOL of HYGIENE &TROPICAL MEDICINE

SMASH

SUBSCRIPTION MODELS FOR ANTIBIOTICS IN HOSPITALS

SMASH survey

Survey finish and contact details

* 49. Would you like to receive the written report of this survey?

◯ Yes

◯ No

* 50. To test the reliability of the SMASH questionnaire, we would like a small subset of participants to repeat the survey approximately one month after their original submission. A second £10 Amazon voucher will be provided for this effort. Please indicate below if you would like to be invited to repeat the survey.

◯ Yes, I would be willing to repeat the SMASH survey

◯ No, thank you, I would not be willing to repeat the SMASH survey

* 51. Please provide your NHS email address below. This will only be used to send you your £10 Amazon voucher, invite you to repeat the survey if you have indicated you would be willing to so do, and send you the survey results, if requested. Your email address will not be used for any other purposes including sending you spam emails, will not be shared with the funder of this study and will not be stored after the end of this study.

Email Address [ ]

Thank you for completing the SMASH survey!

LONDON SCHOOL of HYGIENE & TROPICAL MEDICINE

SMASH

SUBSCRIPTION MODELS FOR ANTIBIOTICS IN HOSPITALS

Appendix II

Supplementary materials and methods:

Study population

The funding model agreement requires consultation with a specialist in infectious diseases or microbiology, who needs to assess the indication for treatment with novel antibiotics. In the UK, there are four infection specialties recognised from the General Medical Council (Infectious Diseases, Medical Microbiology, Medical Virology, Tropical Medicine). Doctors can choose to specialise in any one of the specialties or combinations of two or three of them. For this purpose, they have to complete 2-5 years of general training (foundation and/or core medical training), before becoming registrars (specialty training doctors). Registrar training lasts for another 4 - 5 years as a minimum. At the end, doctors are awarded a Certificate of Completion of Training (CCT) in the speciality or specialities they trained in and are entrusted to practice independently (Consultants). The target population of the SMASH survey was all consultants with a CCT in Infectious Diseases or Medical Microbiology currently working in an NHS acute hospital in England. We defined "currently working" as working in an NHS acute hospital in England for at least 2 Programmed Activities (equivalent to two half days a week). Consultants with a CCT in Infectious Diseases or Medical Microbiology but working exclusively in Virology or Tropical Medicine were excluded.

Survey conduction and administration

This was a cross-sectional survey in November and December 2022, five to six months after the launch of the "subscription-type" funding model, across all NHS acute hospitals. NHS acute hospitals are usually organised in NHS Trusts, which are groups of 1 – 5 affiliated NHS hospitals in geographical proximity, commonly sharing infection clinical and laboratory services. There were 257 NHS hospitals during the study period, organised in 136 NHS Trusts.[1] Groups of all NHS Trusts were assigned to study investigators, who identified all eligible participants in each Trust. Each participant was contacted up to three times and invited to complete the study questionnaire. Responses were not anonymous, as participants provided their professional email addresses for their response to be recorded. Only fully completed responses were recorded. A small financial incentive (10GBP Amazon voucher) was offered to all participants completing the study questionnaire.

Questionnaire design

This was an online-only survey using the SurveyMonkey platform (Momentive Inc., USA). A novel 51-item questionnaire was developed, after consultation with national experts in antimicrobial resistance, antimicrobial pharmacy, health policy and statistical sciences, who assessed draft versions for content, face and criterion validity. The final pre-pilot version was decided after review of all comments received in a dedicated focus group among the primary study investigators (IB, MG, AA). The questionnaire was subsequently piloted among 23 study investigators (infection specialty registrars), who had not seen any previous drafts of the survey. Results and comments from the pilot were discussed in a second dedicated focus group (IB, MG, AA) and the final version of the questionnaire was decided (Appendix I). All questions apart from ten in the final questionnaire were mandatory.

Statistical analysis

Analysis of results was performed in SPSS version 29 (IBM Corp, USA). Categorical variables were expressed as percentages with 95% confidence intervals (CIs), continuous variables were expressed as means with 95% CIs or medians with interquartile ranges (IQRs) as appropriate. 95% CIs were calculated using 10.000 bootstrap samples. Univariable comparisons were made using the Fischer exact test for categorical variables with less than 20 observations; Pearson Chi-squared for categorical

variables with more than 20 observations; McNemars test for paired categorical variables; students t test for normally distributed continuous variables; and Mann-Whitney U test for non-normally distributed continuous variables. For differences across multiple variables, trend tests were initially performed (Chi-squared for categorical variables; Kruskal-Wallis for non-normally distributed continuous variables; ANOVA for normally distributed continuous variables). Individual comparisons were made only if statistically significant trends were established. For interpretation of the results of questions using Likert scales, agreement with the question was defined as the sum of participants who select “agree” or “strongly agree” for their answers. Predefined sensitivity analysis was performed for participants who reported conflicts of interest and for participants who worked with paediatric patients.

Internal consistency of questions addressing similar themes was tested using Cronbach’s alpha.[2] At the end of the survey, participants were invited to complete the questionnaire a second time one month after their original submission. 10% of total responders (arbitrary proportion based on practical and financial feasibility) were randomly selected for this purpose. Test-retest reliability was calculated using Cohen’s kappa for categorical variables.[3]

References

1 NHS Digital. *Estates Returns Information Collection*, <https://digital.nhs.uk/data-and-information/publications/statistical/estates-returns-information-collection> (2022).

2 Tavakol, M. & Dennick, R. Making sense of Cronbach's alpha. *International journal of medical education* **2**, 53 (2011).

3 Leppink, J. & Pérez-Fuster, P. We need more replication research – A case for test-retest reliability. *Perspectives on Medical Education* **6**, 158-164, doi:10.1007/s40037-017-0347-z (2017).
